# Supplementary material for: Burns in Nepal: a participatory, community survey of burn cases and knowledge, attitudes and practices to burn care and prevention in three rural municipalities
Source: BMJ Open. 2020 Feb 28;10(2):e033071. doi: 10.1136/bmjopen-2019-033071 (PMC7050363; doi:10.1136/bmjopen-2019-033071)
Supplement: Author's manuscript [file bmjopen-2019-033071.draft_revisions.pdf]

BMJ Open

BMJ Open is committed to open peer review. As part of this commitment we make the peer review history of every article we publish publicly available.

When an article is published we post the peer reviewers' comments and the authors' responses online. We also post the versions of the paper that were used during peer review. These are the versions that the peer review comments apply to.

The versions of the paper that follow are the versions that were submitted during the peer review process. They are not the versions of record or the final published versions. They should not be cited or distributed as the published version of this manuscript.

BMJ Open is an open access journal and the full, final, typeset and author-corrected version of record of the manuscript is available on our site with no access controls, subscription charges or pay-per-view fees (http://bmjopen.bmj.com).

If you have any questions on BMJ Open's open peer review process please email info.bmjopen@bmj.com

BMJ Open

Burns in Nepal: understanding the patient journey in 3 rural municipalities

Journal:	*BMJ Open*
Manuscript ID	bmjopen-2019-033071
Article Type:	Research
Date Submitted by the Author:	22-Jul-2019
Complete List of Authors:	Phuyal, Kamal; SAGUN: a search for harmony<br>Ogada, Edna; Swansea University, Centre for Global Burn Injury Policy and Research, College of Human and Health Sciences<br>Bendell, Richard; Interburns<br>Price, Patricia; Swansea University; Interburns<br>Potokar, Tom; Swansea University, Centre for Global Burn Injury, Policy and Research, College of Human and Health Sciences; InterBurns
Keywords:	Epidemiology < TROPICAL MEDICINE, Burns, Prevention, Community

SCHOLARONE™
Manuscripts

I, the Submitting Author has the right to grant and does grant on behalf of all authors of the Work (as defined in the below author licence), an exclusive licence and/or a non-exclusive licence for contributions from authors who are: i) UK Crown employees; ii) where BMJ has agreed a CC-BY licence shall apply, and/or iii) in accordance with the terms applicable for US Federal Government officers or employees acting as part of their official duties; on a worldwide, perpetual, irrevocable, royalty-free basis to BMJ Publishing Group Ltd ("BMJ") its licensees and where the relevant Journal is co-owned by BMJ to the co-owners of the Journal, to publish the Work in this journal and any other BMJ products and to exploit all rights, as set out in our licence.

The Submitting Author accepts and understands that any supply made under these terms is made by BMJ to the Submitting Author unless you are acting as an employee on behalf of your employer or a postgraduate student of an affiliated institution which is paying any applicable article publishing charge ("APC") for Open Access articles. Where the Submitting Author wishes to make the Work available on an Open Access basis (and intends to pay the relevant APC), the terms of reuse of such Open Access shall be governed by a Creative Commons licence – details of these licences and which Creative Commons licence will apply to this Work are set out in our licence referred to above.

Other than as permitted in any relevant BMJ Author's Self Archiving Policies, I confirm this Work has not been accepted for publication elsewhere, is not being considered for publication elsewhere and does not duplicate material already published. I confirm all authors consent to publication of this Work and authorise the granting of this licence.

For peer review only

Title: *Burns in Nepal: understanding the patient journey in 3 rural municipalities*

Authors:

Kamal Phuyal[1], Edna Ogada[2], Richard Bendell[3], Patricia Price [2,3], Tom Potokar [2,3]

1. SAGUN: a search for harmony; Kupandol, Lalitpur, Nepal
2. Centre for Global Burn injury Policy and Research, Swansea University
3. InterBurns, Welsh Centre for Burns and Plastic Surgery, Swansea

Corresponding author:

Professor Patricia Price,

Centre for Global Burn injury Policy and Research,

College of Human and Health Sciences, Room 26 Haldane Building

Swansea University, Singleton Park, Swansea SA2 8PP

Email: p.e.price@swansea.ac.uk

Keywords:

Burns/epidemiology: Burns/prevention: Community participation: Survey: First Aid

Abstract

Objectives: As part of an on-going, long-term project to co-create prevention strategies in Nepal, we undertook a community-based survey to gather baseline data to share and discuss with the local community, use as a basis for a co-created prevention strategy and then monitor changes over time. This paper reports on the method and outcomes of the initial survey.

Setting: Community based: 3 Rural Municipalities in Nepal

Participants: 1305 households were approached; the head of 1279 households participated, giving a response rate of 98%. In 90.3% of cases, the head of the household was male.

Results: We found that 2.7% (CI: 1.8, 3.7) of 1279 households, from 3 representative municipalities, reported at least one serious burn in the previous 12 months. Whilst only 4 paediatric and 10 adult cases in the previous 12 months reached hospital care, the impact on the lives of those involved was profound; the data must be interpreted with caution due to the potential for memory bias and does not include mortality data for burn injury. These data indicate that the self-referral patterns are appropriate and that a range of first aid behaviours are used, many of which are appropriate for the local context whilst a few may be potentially harmful.

Conclusion: The study highlights the importance of very local knowledge when interpreting the data and how sharing the data in an easily digestible format, alongside community participation at all

For peer review only - http://bmjopen.bmj.com/site/about/guidelines.xhtml

stages, can inform the co-creation of locally appropriate prevention strategies. As a next stage we are developing a tool-kit of prevention strategies to evaluate in the country, together with education and training from basic to advanced levels.

Article Summary: Strengths

- Detailed and sustained work with the local community to ensure enhanced community participation in the survey
- The use of info-graphics to overcome language and knowledge barriers when presenting the findings back to the community
- Co-creation of sustainable prevention strategies with the local communities

Limitations

- Only one person with a burn injury per household was included in the survey.

For peer review only

For peer review only - http://bmjopen.bmj.com/site/about/guidelines.xhtml

Introduction

The World Health Organisation has repeatedly tried to raise awareness of the fact that the burden of burn injury, from a global perspective, is borne by low and middle-income countries [1,2]. Patients in these countries continue to endure an extremely high level of suffering, with the rate of child deaths from burns being over 7 times higher in lower resource settings than in the western environment[3,4]; other recent publications[5] have confirmed that the greater burden of mortality and morbidity related to burn injury is borne by the world's poor, residing mostly in LMICs.

To bring about lasting changes in resource poor countries, it is vital that we have a detailed understanding of the current, local context including epidemiology, the availability of educational/prevention programmes and the ability of people in rural/remote areas to access vital first aid treatment. This paper focuses on the first part of a major initiative to bring about change using an Implementation Science approach, based in Nepal. Our experiences in the area have highlighted that the local communities feel that they are frequently being asked to participate in surveys – yet feel that nothing changes. So, although it is important to fully understand the baseline situation so that we can evaluate whether our intervention has been effective, we have also sought ways of presenting our findings to the local community that are easy to understand across languages. The focus has been to highlight the ways in which people access burn care by understanding their pathway from injury to treatment as a first step in co-creating prevention strategies that are locally relevant and implementable.

The Annual Report from the Department of Health and Population for Nepal, covering the fiscal year 2016-17, recorded 55,090 burn or scald injuries nationally[6]. Population statistics for the same time period were 28.9M, giving an incidence rate of 0.19% of patients with an injury sufficiently severe to seek help from an outpatient facility where data were recorded. Using the ICD codes T200 – T301, for inpatients, 1710 cases were reported with 1006 related to those below the age of 19, and 586 under the age of 5 (81.7% of these were aged 1-4). In the same time period there were 46 deaths (31 F, 15 M) – although this does not include deaths from catastrophic events or from those who were sent straight to the mortuary.

Whilst these studies start to give us an indication of the scale of the problem, they do not give us information about the ways in which people sought help, what they did immediately after the injury, what local knowledge exists to provide first-aid and what prevention programmes are available locally. In order to ensure that we could fully evaluate our intervention, we needed to conduct a more in-

For peer review only - http://bmjopen.bmj.com/site/about/guidelines.xhtml

depth community based survey so that we could develop and co-create (with community stakeholders) a community prevention and first aid strategy. This paper presents the findings of these baseline phases of the study.

Methods

This three-phased study uses a participatory action research design to develop a prevention and first aid strategy at the rural municipality level (RM) (n= 460 RMs in Nepal). The first phase included a community sensitization and preparation plan, followed by baseline data collection. As the prevention strategies need to be developed at the rural municipality level, baseline data were collected in one randomly selected municipality in each of three districts, Ama Chhodingmo Rural Municipality (ARM) in Rasuwa, Indrasarowar Rural Municipality (IRM) in Makawanpur and Laxminiya Rural Municipality (LRM) in Dhanusha. These districts were drawn from three traditionally noted geographic regions of Nepal; Terai (flatland), Hills, and Himalayan regions. This purposive selection was used to ensure a degree of diversity in the communities studied.

The study population was all the people living in those rural municipalities, regardless of socio-demographic factors. A sample of 1305 households was selected from a population of 47,642, based on the WHO formula[7], adjusted to account for the relative population size in each district. Household selection was completed using a systematic sampling approach drawing from a listing of the total number of households in each RM, formulated during the sensitisation preparation phase at each municipality office, when detailed discussions took place with local stakeholders.

Rasuwa district is located north of Kathmandu. It is a district of natural resources, particularly Langtang and Gosaikunda regions, which are well known for their natural beauty and trekking routes, with a high potential for eco-tourism businesses. However, poor roads, poor access to higher education and poverty have hindered the development of the district. Ama Chhodingmo is located in the mountain region, bordering China to the north, and contains both natural and cultural diversity. The landscape in this area makes road construction very difficult, and most of the northern wards (in higher up areas) lack access to vehicle-friendly roads. Rasuwa itself is accessible by bus from Kathmandu via the Pasang Lhamu highway, with its headquarters (Dhunche) about 120 km from the capital.

Makawanpur is a hilly district, located on the southern border of Kathmandu. Despite bordering Kathmandu valley, many wards in Makwanpur remain inaccessible by road. The main livelihood for

For peer review only - http://bmjopen.bmj.com/site/about/guidelines.xhtml

the majority of the population is subsistence farming. The district includes within its borders various agro-climatic zones: valleys, plains, and mid-to-high hills. High rates of unemployment have driven a surge of migration - particularly of younger people - to Kathmandu and abroad. Indrasarowar rural municipality, selected for this project, is located in the north eastern part of the district in which the majority of the population belong to Tamang ethnic group.

Dhanusha is one of the southern Terai districts of Janakpur Zone and is an agriculture dependent district. Janakpurdham city is the capital of State 2 where this district is located and one of the commercial centres in the Terai region. It is also the administrative hub of the state. Laxminiya rural municpality is located around 5 km from the major city of Janakpur; the settlement straddles a highway, allowing for some road access. The main livelihood is subsistence farming. The majority of people in this rural municipality belong to Madhesi middle caste (i.e. Yadav, Sudhi, Lohar, Sonar) and Maithili is their main language. This is the most densely populated area included in the study.

Initial discussions with the key stakeholders included 15 Key Informant Interviews (KIIs) and 19 Focus Group Discussions (FGDs) with Female Community Health Volunteers (FCHV), teachers, social workers and health care staff. This important first stage of the study allowed the researchers to develop their understanding of the general context of the burn injuries. Participant observation sessions (n=13) that focused on food storage and cooking practices, together with social maps and seasonal calendars, were used to augment the narrative on burn injuries, their prevention and management in each of the rural municipalities. Based on this initial work items for the survey were prepared both in Nepali and English and reviewed by 5 local experts (including Sagun board members and university staff) to enhance their validity. The data collection tool asked households to report on any burn injury that required medical attention in the previous 12 months.

Survey data was collected using a structured questionnaire to collect data on social demographic and household descriptors; incidences of burn injury meeting the case definition (i.e., a burn injury serious enough to require medical attention), first aid received and health seeking behaviour after the burn injury. The questionnaire was pre-tested in Mangaltar of Kavre district, where it was piloted on 15 households; only minor changes were required at the end of this phase. The final version of the survey tool was developed into an electronic format (CSPro software) in order to assist with the logistics of collecting data in remote areas, where smart phones are used routinely despite the geographic remoteness.

For peer review only - http://bmjopen.bmj.com/site/about/guidelines.xhtml

All enumerators were trained on data collection using an electronic version of the form on smart phones to ensure consistency of use and reduce human error. The training included a day on theory and a day on practical issues, followed up by 3 supervised community-based training opportunities resulting in 3-5 full days of supervised training. All enumerators were instructed to call at the selected households up to 3 times until someone was present to complete the questionnaire: after 3 attempts no further attempt was made to contact the household members.

Three introductory visits were organised in each RM to discuss the details of the research proposal with representatives of the local government, health care staff and other relevant stakeholders at the RM level. A letter of approval was provided by each of the relevant local governments. The research proposal was reviewed and approved by Nepal Health Research Council (NHRC-Reg. no.348/2018). Survey data was recorded and analysed using IBM SPSS version 23.

The majority of the analysis was descriptive in nature and involved summarising findings by region, gender, type of burn, initial first aid and final destination of health service need. The data are presented for the whole cohort, as well as by region using pictorial schematics that indicate the total experience for each individual person with a burn injury. The unit of analysis was taken to be 'the household'.

Patient and Public Involvement

The public and former patients were involved in the design of the study through interviews with key personnel, by allowing the researchers to observe their everyday practices (for example, routine cooking and food storage practices) and through community focus groups (n=19). The key stakeholders involved included political leaders, health post workers, household members (including burn survivors), women's groups, and school teachers. These same groups were involved in the direct dissemination of the community findings using info-grams, and case-based narratives to develop locally relevant, and locally owned awareness campaigns, prevention strategies and educational materials to support appropriate first-aid practices.

Results

1305 households were included in the original sampling frame; data were recorded from 1279 (98% of the initial sample). In the 12 months preceding the survey, 2.7% of the households reported at least one serious burn injury (see Table 1). Of the three RM's, Laxminiya reported 4 of every 100 households experiencing a burn injury, Indrasarowar reported 1:100, while Ama Chhodingmo reported 3:100. More women were affected, scalds being the most frequent cause of injury and the injury was most

For peer review only - http://bmjopen.bmj.com/site/about/guidelines.xhtml

likely to happen at home; more injuries were reported in adults than children over the 12-month time frame.

Table 2 presents descriptive data on knowledge and practices on burn prevention, first aid and health seeking behaviour in all 1279 household. Exposure to prevention education is limited, with only one in ten households self-reported exposure to education or information about first aid treatment for burns. Two-thirds of the households indicated that they believe that most burns are preventable, with a significantly greater proportion (74%) in Laxminiya; interestingly, this is where the rates for burn injury in the last 12 months were the highest.

The most frequent response to first aid action was the use of water when a family member is scalded (27.4%), with the application of lotion as the lowest response (5.7%). However, Table 2 also outlines regional variations with Laximiniya reporting the highest use of water (38.6%) whereas the highest response in Indrasarowar is to apply tomato, and the highest response in Ama Chhodingmo is to use a range of other options (including mango leaf paste, cow dung, mud, and herbs). Households in Ama Chhodingmo also reported that 28.2% say they would not know what to do in such circumstances.

Forty percent would take off clothing if they caught fire (40.4%); assuming it was available, 3 in 10 households reported they would either pour water onto the victim or jump into water. Only 3 in every 100 households reported that they would stop, drop and roll, while about 1 in 10 reported they would smother the flames with a cloth. One out of every 10 households indicated they would not know what to do. These overall figures again hide substantial differences in regional responses (Table 2).

The majority of Households responded that they would take a family member to hospital following a major burn (73.4%) or to a primary healthcare facility for a minor burn (57.5%). There were regional differences, with only a little over half of households in Ama Chhodingmo reporting they would take a family member to hospital immediately after a large or major burn (55.6%). This could be a reflection of lack of access in terms of resources - financial and otherwise, or physical distance to secondary or tertiary level services. One third of all households reported that they would manage minor burn incidents at home immediately after the incident, which was consistent across the three sites.

The data relating the experiences of those injured in the previous 12 months have been summarised in an info-gram (Figure 1) which illustrates patient journeys from initial injury to treatment; each identified case from the data base has been given an icon to reflect whether they are male or female,

For peer review only - http://bmjopen.bmj.com/site/about/guidelines.xhtml

and an adult or a child. The left hand side of the icon reflects the type of burn injury, with the right hand side reflecting their rural municipality. For example, Case A is a female child from Laximinya, who experienced a flame burn, was treated initially with an alternative form of first aid (tube/lotion) and was transferred by ambulance 3-4 days after injury and then spent 35 days in hospital.

-----Figure 1 about here-------------------

Figure 1 shows that only 5 out of 34 burns received appropriate first aid in the form of cooling the wound prior to applying alternative methods for first aid, with 10 burns receiving no formal medical treatment. One in 4.4 burn victims applied some form of tube based lotion or medicine as a first aid measure indicating the availability of some form of 'over the counter' burn remedy. Minor burns that can be managed at health posts or pharmacies are filtered appropriately by self-referral, while major or serious burns present to secondary or tertiary hospitals. The data indicate that most of the serious burn cases requiring admission are disproportionately experienced by women/girls; in all cases they are linked to cooking activities. Figure 2, which summarises the economic and social costs for the 24 individuals who received formal treatment (10 at primary and 14 at secondary/tertiary level), shows that more than half reported having to borrow money or sell assets to cover the costs of their treatment. One in four suffered a disability that resulted in full dependence to perform one or more daily tasks.

-------Figure 2 about here---------------

Discussion

The average time spent at primary vs secondary/tertiary level in the patient journey clearly demonstrates that community members appropriately self-select for medical treatment at the primary and secondary/tertiary level of care for burn injuries. Our data suggest a self-report of serious burn injury, requiring secondary or tertiary care, was reported in 2.7% of the population; 9.72% in Dhanusa, 1.29% in Rasuwa and 4.25% in Makwanpur, when all presentations to a health care professional are included (i.e., including primary health level). The higher than anticipated figures, based on the Annual Report[6], reflect a substantial number of households that have experienced a serious burn, many of whom needed hospital care and will need on-going management for their pain, disabilities and limitations in function, and ability to be self-sufficient.

Together with the background information collected in the initial phases of the study, the survey provides great insight into the risk factors and prevailing knowledge of burn injury at the rural municipality level. Presenting this information in a simple visual format to local stakeholder groups

For peer review only - http://bmjopen.bmj.com/site/about/guidelines.xhtml
For peer review only

enables informed discussion and supports the co-creation approach that will empower local communities to implement prevention activities. Our ability to plot the experiences of individuals from initial injury through to length of hospital stay, will allow local health teams to work in tandem with local communities to fully explain the importance of initial first aid and the consequences of delayed (or inappropriate) treatment. The use of info-grams, to support patient understanding of health messages[8], has enabled us to summarise the data in an easily digestible format and allows us to work with a wide range of stakeholders. Although some translation will be required, the emphasis on imagery should support us to disseminate messages to health care professionals across the country. Initial work on scoping out a prevention strategy has highlighted the importance of this very local knowledge when interpreting the data, and of info-grams as a focal point for meaningful discussions when co-creating a context-relevant prevention strategy.

These data suggest that the key areas for prevention would be cooking within the home setting, particularly for women and children. Local knowledge will be extremely important in identifying how this needs to be built into a local program. First aid messages need to stress the importance of cool clean water as an initial treatment, when it is available (particularly for scald and flame burns); however, local programs will need to reflect on what is locally available and differentiate between acceptable, alternative cooling options and those that must be avoided (e.g., mud). When clothing catches fire, household members need to be aware of the stop, drop and roll method and prioritise that no additional body parts (or other individuals or items) catch fire in the process.

This was a robustly designed study with a strong emphasis on using local knowledge to inform the content of the data collection tool, and substantial emphasis was placed on spending time with the local communities to ensure their full involvement. However, it is important not to over interpret the findings of the survey at either district or national level; any method that relies on recall is subject to memory bias. In this study, the proportional distribution of households recruited roughly reflected the relative population sizes of the RM's in each district.

Only one member of each household, even in households with multiple burn injuries, was interviewed about the incident; the individual selected was the one with the most serious injury meeting the case definition. This approach may have resulted in a potential underestimation of burn injuries and any new studies should consider interviewing all those with a serious burn injury.

For peer review only - http://bmjopen.bmj.com/site/about/guidelines.xhtml

Those involved in the initial stages of the project, in collaboration with the research team, are already working to co-create appropriate prevention strategies including a wide range of locally relevant activities, where the local community propose ideas that would make a real difference for their own communities. This is an important initial step in developing locally relevant and locally owned prevention strategies to help reduce the incidence of burns, whilst ensuring that those with an injury receive the most appropriate first aid and seek appropriate care as quickly as possible.

Acknowledgements

We would especially like to acknowledge the support of Mr Bindeshwor Yadav, Chairperson, Laxminiya Rural Municipality, Dhanusha; Mr Buchung Tamang, Chairperson, Ama Chhodingmo Rural Municipality, Rasuwa; and Mr Jivan Lama, Chairperson, Indrasarowar Rural Municipality, Makawanpur.

With thanks to Arjun Bhattarai for his support in developing the online tool for data capture and support with the sampling frame.

The project team would like to express their heartfelt thanks to everyone who took the time to participate in the development of the survey, the survey itself and to the local SAGUN team members who are so enthusiastic to bring about change and improvements in their local communities in Nepal.

Contribution of authors

Kamal Phuyal: Local lead for SAGUN in Nepal and the local research contact for the survey

Edna Ogada: Research assistant with responsibility for assisting with the ethical approval process, organising and analysing the data

Richard Bendell: Program manager for InterBurns, supported the logistics for the project and the design of the data collection tool

Patricia Price: Academic lead and responsible for writing the paper

Tom Potokar: Grant holder and Chief Investigator.

All authors have reviewed the paper prior to submission.

Competing Interests

There are no competing interests for any of the authors.

For peer review only - http://bmjopen.bmj.com/site/about/guidelines.xhtml

Funding

This project is funded by the National Institute for Health Research (NIHR), NIHR Global health Research Group on Burn Trauma, Grant Reference 16/ 137/ 110. The views expressed are those of the author(s) and not necessarily those of the NIHR or the Department of Health and Social Care.

Data Sharing

Data will be available from NIHR Open Data supplied by NIHR Centre for Business Intelligence, Crown copyright and database licensed under the Open Government License (http://www.nationalarchives.gov.uk/doc/open-government-licence/version/3/): Global Health Research (in development). Data will also be archived at Swansea University.

References

1. Peck M, Molnar J & Swart D. A global plan for burn prevention and care. *Bulletin of the World Health Organisation* 2009;**87**: 802-803 doi: 10.2471/BLT.08.059733
2. WHO Factsheet on Burns: March 2018 (accessed May 7 2019) available at http://www.who.int/news-room/fact-sheets/detail/burns
3. Mathers CD, Bernard C, Moesgaard Iburg K, *et al*. 2003. Global Burden of Disease in 2002: data sources, methods and results (Version 2). *Global Programme on Evidence for Health Policy Discussion* Paper No.**54**. World Health Organisation; Geneva http://www.who.int/healthinfo/paper54.pdf
4. Sengoelge M, El-Khatib Z & Laflamme L. The global burden of child burn injuries in light of country level economic development. *Preventive Medicine Reports* 2017: **6**: 115–120 http://dx.doi.org/10.1016/j.pmedr.2017.02.024
5. Stokes, MAR & Johnson WD. Burns in the Third World: an unmet need. *Annals of Burns Fire Disasters*. 2017 Dec 31; **30**(4): 243–246.
6. Ministry of Health and Population. *Annual Report,* 2016. Department of Health Services, Kathmandu, Nepal
7. Sethi D, Habibula S, McGee K, *et al*. (Eds) *Guidelines for Conducting Community Surveys on Injuries and Violence.* 2004 World Health Organisation, Geneva ISBN: 92 4 154 648 4
8. Barros IM, Alcantara TS, Mesquita M *et al* The use of pictograms in health care: a literature review. *Res Social Admi Pharm* 2014, Sept-Oct; **10** (5): 704-19 http://doi:10.1016/j.sapharm.2-13.11.002

For peer review only - http://bmjopen.bmj.com/site/about/guidelines.xhtml

Table 1: Prevalence for burn injury in three rural municipalities in Nepal, 2017- 2018 (n= 1279 Households)

**% responses**	Indrasarowar, Makwanpur (n=415)	Laxminiya, Dhanusha (n=630)	Ama Chhodingmo, Rasuwa (n=234)	**Total**	**95 % CI**
at least one burn injury	1.2	3.5	3.0	2.7	1.8,3.7
**who was burned**					
children	0.5	1.4	1.3	1.1	0.6, 1.8
adults	0.7	2.1	1.7	1.6	1.0,2.4
women	0.7	2.4	1.7	1.7	1.1, 2.6
men	0.5	1.1	1.3	0.9	0.5, 1.6
**cause of burn**					
flames	0.0	1.7	0.4	0.9	0.5,1.6
scalding	1.0	1.6	1.7	1.4	0.8,2.2
other mechanisms	0.2	0.2	0.9	0.3	0.1,0.8
**where the burn injury occurred**					
at home	1.2	3.5	2.1	2.5	1.7,3.5
away from the home	0.0	0.0	0.9	0.2	0.0,0.7
**location of injury**					
head and neck	0.0	0.5	0.0	0.2	0.0,0.7
limbs	1.0	3.0	2.1	2.2	1.5,3.1
trunk	0.2	0.0	0.9	0.2	0.0,0.7
**whether the injury occurred during cooking**					
while cooking	1.2	3.5	2.1	2.5	1.7,3.5
other activities	0.0	0.0	0.0	0.0	0.0

For peer review only

For peer review only - http://bmjopen.bmj.com/site/about/guidelines.xhtml

Table 2: Burn prevention, first aid and health seeking behaviour in Nepal, 2017- 2018

% responses	Indrasarowar, Makwanpur(n=415)	Laxminiya, Dhanusha(n=630)	Ama Chhodingmo, Rasuwa(n=234)	Total	95 % CI
**burn prevention**					
education on prevention	13.5	10.8	6.8	10.9	9.3,12.8
believe most burns are preventable	52.3	73.8	47.4	62.0	59.3,64.7
believe most people with burns die as a result of their injury	68.0	82.2	70.1	75.4	72.9,77.7
believe that burns can cause bad scars	85.1	96.5	85.5	90.8	89.1,92.3
**first aid**					
received education	15.7	10.5	5.6	11.3	9.6,13.1
use water	18.3	38.6	13.2	27.4	24.9,29.9
tomato	39.3	5.9	14.1	18.2	16.1,20.4
potato paste	0.0	25.4	0.0	12.5	10.7,14.4
aloe vera	20.0	2.5	1.7	8.1	6.6, 9.7
honey	0.0	13.3	0.0	6.6	5.3,8.1
lotion/tube medicine	5.1	4.6	9.8	5.7	4.5,7.1
other (in order of frequency mango leaf paste, cow dung, mud, herbs, various oils and plants)	5.5	5.9	32.9	10.7	9.1,12.5
would not know what to do	11.8	3.8	28.2	10.9	9.2,12.7
**Clothing catching on fire**					
stop, drop and roll	1.7	5.9	0.0	3.4	2.5,4.6
smother flames with a cloth	13.0	16.7	3.4	13.1	11.3,15
jump in water	7.7	11.6	1.3	8.4	7,10.1
take off the clothing	28.4	53.8	25.6	40.4	37.7,43.2
pour water	34.7	8.7	31.6	21.3	19.1,23.7
would not know what to do	13.0	1.7	36.3	11.7	10, 13.6
**Health seeking behaviour (large or major burn)**					
hospital immediately after incident	67.7	83.8	55.6	73.4	70.9,75.8
primary health care facility* immediately after incident	29.2	15.5	40.6	24.6	22.2,27.0
**Health seeking behaviour (minor burn)**					
manage at home	33.7	37.6	34.6	35.8	33.2, 38.5
primary health care facility*	58.3	58.6	53.0	57.5	54.7,60.2
hospital	1.7	2.4	5.6	2.7	(1.9, 3.8)

*Mainly health post but some pharmacy

For peer review only - http://bmjopen.bmj.com/site/about/guidelines.xhtml

What did we know already?

- The majority of burn injuries occur in low-middle income countries
- Nepal Government reported a prevalence of 0.19% of injuries sufficiently serious to seek medical help for 2016-17

What do we know now?

- Very detailed work with local communities is required before a community survey can take place
- A co-created community survey reveals a prevalence of 2.7% in randomly selected districts from 3 rural municipalities
- Very local knowledge is needed to interpret the data in a meaningful way
- Presenting the data in a digestible format is important for local communities to engage in co-creating prevention strategies

For peer review only

For peer review only - http://bmjopen.bmj.com/site/about/guidelines.xhtml

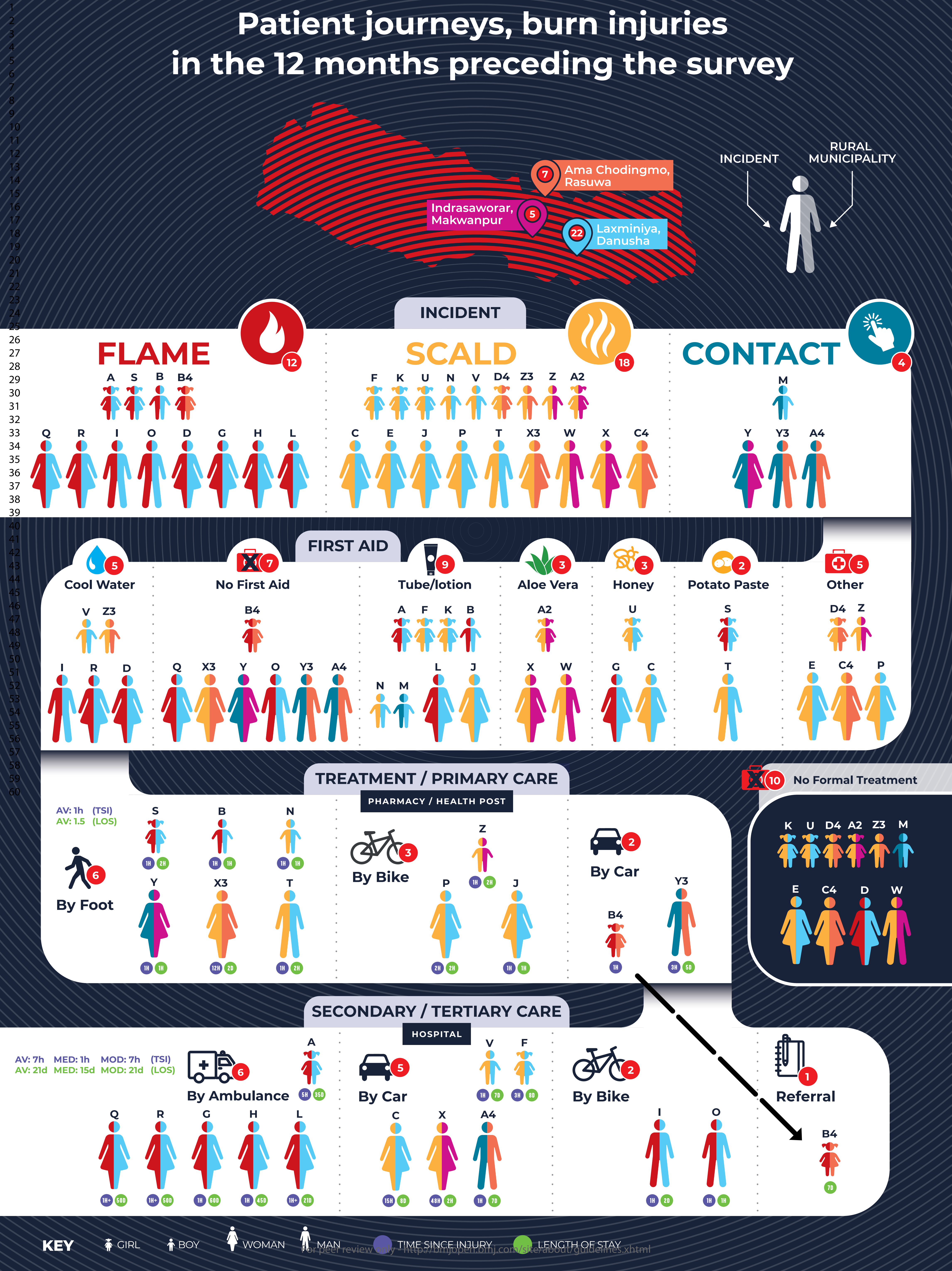

For peer review only - http://bmjopen.bmj.com/site/about/guidelines.xhtml

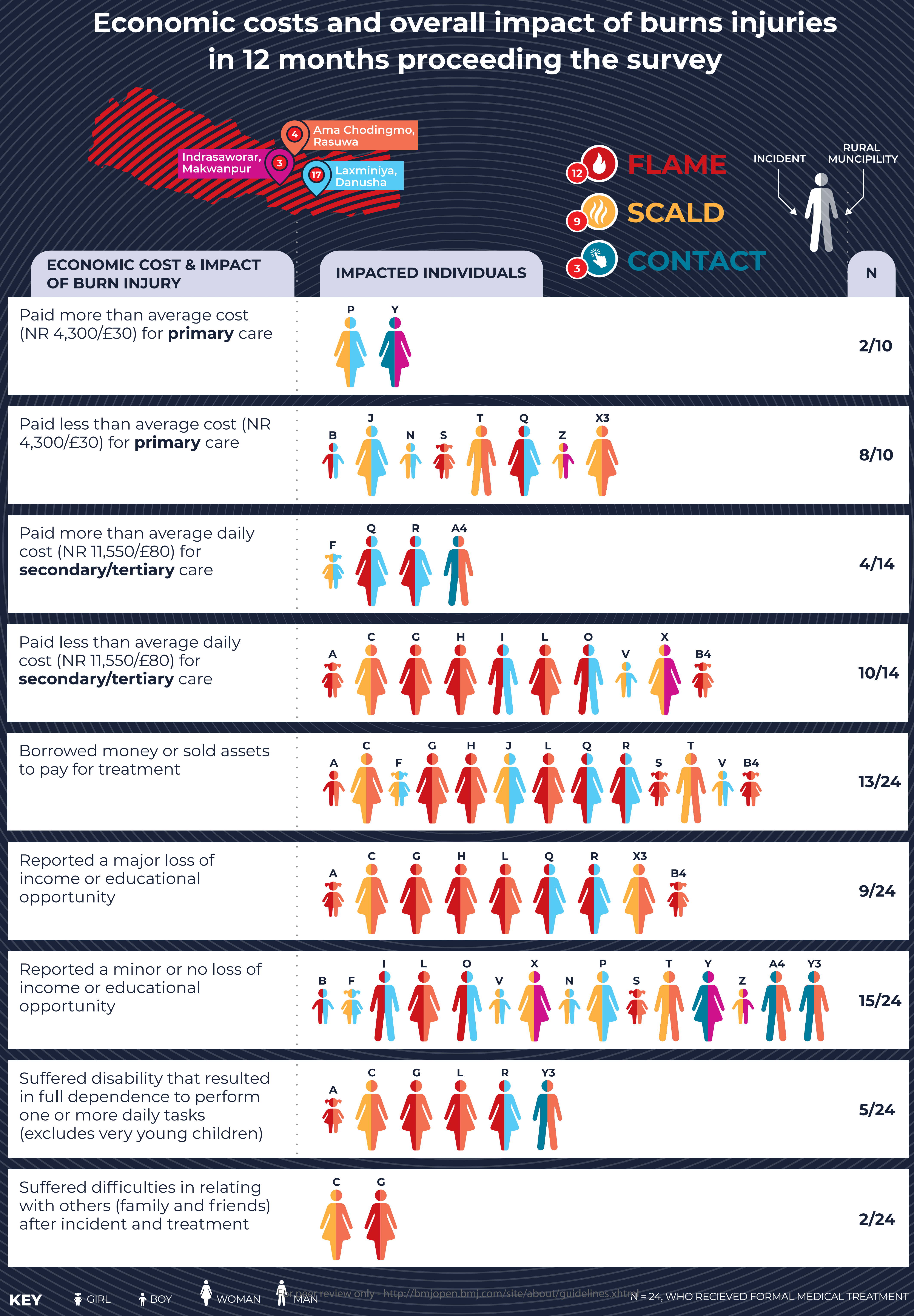

For peer review only - http://bmjopen.bmj.com/site/about/guidelines.xhtml

BMJ Open

Burns in Nepal: a participatory, community survey of burn cases and knowledge, attitudes and practices to burn care and prevention in 3 rural municipalities

Journal:	*BMJ Open*
Manuscript ID	bmjopen-2019-033071.R1
Article Type:	Original research
Date Submitted by the Author:	29-Oct-2019
Complete List of Authors:	Phuyal, Kamal; SAGUN: a search for harmony<br>Ogada, Edna; Swansea University, Centre for Global Burn Injury Policy and Research, College of Human and Health Sciences<br>Bendell, Richard; Interburns<br>Price, Patricia; Swansea University; Interburns<br>Potokar, Tom; Swansea University, Centre for Global Burn Injury, Policy and Research, College of Human and Health Sciences; InterBurns
**Primary Subject Heading**:	Public health
Secondary Subject Heading:	Epidemiology
Keywords:	Burns, Prevention, Community

SCHOLARONE™
Manuscripts

I, the Submitting Author has the right to grant and does grant on behalf of all authors of the Work (as defined in the below author licence), an exclusive licence and/or a non-exclusive licence for contributions from authors who are: i) UK Crown employees; ii) where BMJ has agreed a CC-BY licence shall apply, and/or iii) in accordance with the terms applicable for US Federal Government officers or employees acting as part of their official duties; on a worldwide, perpetual, irrevocable, royalty-free basis to BMJ Publishing Group Ltd ("BMJ") its licensees and where the relevant Journal is co-owned by BMJ to the co-owners of the Journal, to publish the Work in this journal and any other BMJ products and to exploit all rights, as set out in our licence.

The Submitting Author accepts and understands that any supply made under these terms is made by BMJ to the Submitting Author unless you are acting as an employee on behalf of your employer or a postgraduate student of an affiliated institution which is paying any applicable article publishing charge ("APC") for Open Access articles. Where the Submitting Author wishes to make the Work available on an Open Access basis (and intends to pay the relevant APC), the terms of reuse of such Open Access shall be governed by a Creative Commons licence – details of these licences and which Creative Commons licence will apply to this Work are set out in our licence referred to above.

Other than as permitted in any relevant BMJ Author's Self Archiving Policies, I confirm this Work has not been accepted for publication elsewhere, is not being considered for publication elsewhere and does not duplicate material already published. I confirm all authors consent to publication of this Work and authorise the granting of this licence.

For peer review only

Title: *Burns in Nepal: a participatory, community survey of burn cases and knowledge, attitudes and practices to burn care and prevention in 3 rural municipalities*

Authors:

Kamal Phuyal[1], Edna Ogada[2], Richard Bendell[3], Patricia Price [2,3], Tom Potokar [2,3]

1. SAGUN: a search for harmony; Kupandol, Lalitpur, Nepal
2. Centre for Global Burn injury Policy and Research, Swansea University
3. InterBurns, Welsh Centre for Burns and Plastic Surgery, Swansea

Corresponding author:

Professor Patricia Price,

Centre for Global Burn injury Policy and Research,

College of Human and Health Sciences

Room 26 Haldane Building

Swansea University

Singleton Park

Swansea SA2 8PP

Email: p.e.price@swansea.ac.uk

Keywords:

Burns/epidemiology: Burns/prevention: Community participation: Survey: First Aid

For peer review only

Abstract

Objectives: As part of an on-going, long-term project to co-create burn prevention strategies in Nepal, we undertook a community-based survey to gather baseline data to share and discuss with the local community, use as a basis for a co-created prevention strategy and then monitor changes over time. This paper reports on the method and outcomes of the baseline survey and demonstrates how the data are presented back to the community.

Setting: Community based in 3 Rural Municipalities in Nepal.

Participants: 1305 households were approached: the head of 1279 households participated, giving a response rate of 98%. In 90.3% of cases the head of the household was male.

Results

We found that 2.7% (CI: 1.8, 3.7) of 1279 households, from 3 representative municipalities, reported at least one serious burn in the previous 12 months: a serious burn was defined as one requiring medical attention and/or inability to work or do normal activities for 24 hours. Whilst only 4 paediatric and 10 adult cases in the previous 12 months reached hospital care, the impact on the lives of those involved was profound. Only one patient was referred on from primary to secondary/tertiary care; the average length of hospital stay for those presenting directly to secondary/tertiary care was 21 days. A range of first aid behaviours were used, many of which are appropriate for the local context whilst a few may be potentially harmful (e.g., the use of dung).

Conclusion

The participatory approach used in the study ensured a high response rate. Local partners have been used to help understand the data in context. We have used the results to prepare info-graphics that will now be used to inform the co-creation of locally appropriate prevention strategies.

For peer review only

Article Summary:

Strengths

- Detailed and sustained work with the local community to ensure enhanced community participation in the survey
- Tracking individual patient journeys from injury to final destination for burn care
- The use of info-graphics to overcome language and knowledge barriers when presenting the findings back to the community.

Limitations

- Only one person with a burn injury per household was included in the study.

For peer review only - http://bmjopen.bmj.com/site/about/guidelines.xhtml

Introduction

The World Health Organisation has repeatedly tried to raise awareness of the fact that the majority of the burden of burn injury, from a global perspective, is borne by low and middle-income countries [1,2]. Other recent publications[3] have confirmed that the greater burden of mortality and morbidity related to burn injury is borne by the world's poor, residing mostly in LMICs. Patients in these countries continue to endure an extremely high level of suffering, with the rate of child deaths from burns being over 7 times higher in lower resource settings than in the western environment[4,5]. Mongolia has a particularly high rate of child burn injury as identified by The Global Burden of Disease study in 2013[5]; detailed community studies, conducted in Ulaanbaatar, have supported this finding and shown that 27% (N=247/900) had a history of burn injury[6]. A recent systematic review[7] found eight epidemiological studies based in Nepal and concluded that burn injuries are the leading cause of morbidity and mortality in the country. The authors conclude that the limited data explains the lack of effective intervention programmes in the area.

The Annual Report from the Department of Health and Population for Nepal, covering the fiscal year 2016-17, recorded 55,090 burn or scald injuries nationally[8]. Population statistics for the same time period were 28.9M, giving an incidence rate of 0.19% of patients with an injury sufficiently severe to seek help from an outpatient facility where data were recorded. Data from public hospitals (92% response rate) and private facilities (47.2% response rate) submitted as part of the Annual Report[8] and using the ICD codes T200 – T301 for inpatients, 1710 cases were reported with 1006 related to those below the age of 19, and 586 under the age of 5 (81.7% of these were aged 1-4). In the same time period there were 46 deaths (31 F, 15 M) – although this does not include deaths from catastrophic events or from those who were sent straight to the mortuary.

To bring about lasting changes in resource poor countries, it is vital that we have a detailed understanding of the current, local context including epidemiology, the availability of educational/prevention programmes and the ability of people in rural/remote areas to access vital first aid treatment in order to plan appropriate interventions. This paper focuses on the first part of a major initiative to address these questions and bring about change using an Implementation Science approach, based in Nepal. Whilst previous studies start to give us an indication of the scale of the problem, they do not give us information about the ways in which people sought help, what they did immediately after the injury, what local knowledge exists to provide first-aid and what prevention programmes are available locally. In order to ensure that we could fully evaluate our intervention, we needed to conduct a more in-depth community based survey so that we could

For peer review only - http://bmjopen.bmj.com/site/about/guidelines.xhtml

develop and co-create (with community stakeholders) a community prevention and first aid strategy. This paper presents the findings of the baseline phase of the study. The specific objectives, related to selected areas of Nepal, were to: –

i. record the incidence and causes of burn injury in 2017-18,

ii. explore the knowledge, attitudes and practices of the community regarding burn care and prevention.

Methods

The ongoing study uses a participatory action research design to develop a prevention and first aid strategy at the rural municipality level (RM) (n= 460 RM in Nepal). This first phase included a community sensitization and preparation plan, followed by baseline data collection. As the prevention strategies need to be developed at the rural municipality level, baseline data was collected in one randomly selected municipality in each of three districts, Ama Chhodingmo Rural Municipality (ARM) in Rasuwa, Indrasarowar Rural Municipality (IRM) in Makawanpur and Laxminiya Rural Municipality (LRM) in Dhanusha. These districts were drawn from three traditionally noted geographic regions of Nepal, Terai (flatland), Hills, and Himalayan regions. This purposive selection was used to ensure a degree of diversity in the communities studied, whilst representing the geo-political areas that any national policy would need to address.

The study population was all the people living in those rural municipalities, regardless of socio-demographic factors. A sample of 1279 households was selected from a population of 47,642, based on the WHO formula[9], adjusted to account for the relative population size in each district. Household selection was completed using a systematic sampling approach drawing from a listing of the total number of households in each RM, formulated during the sensitisation preparation phase at each municipality office, when detailed discussions took place with local stakeholders.

Rasuwa district is located north of Kathmandu. It is a district of natural resources, particularly Langtang and Gosaikunda regions, which are well known for their natural beauty and trekking routes, with a high potential for eco-tourism businesses. However, poor roads, poor access to university education and poverty have hindered the development of the district. Ama Chhodingmo is located in the mountain region, bordering China to the north, and contains both natural and cultural diversity. The landscape in this area makes road construction very difficult, and most of the northern wards (in higher up areas) lack access to vehicle-friendly roads. Rasuwa itself is accessible by bus from Kathmandu via the Pasang Lhamu highway, but the administrative headquarters (Dhunche) are

For peer review only

about 120 km from the capital. Over 98% of the population are Hill Ethnic Groups or Tamang; this is one of the indigenous inhabitant groups of Nepal, with their own distinct language, living in scattered hill villages.

Makawanpur is a hilly district, located on the southern border of Kathmandu. Despite bordering Kathmandu valley, many wards in Makwanpur remain inaccessible by road. The main livelihood for the majority of the population is subsistence farming. The district includes within its borders various agro-climatic zones: valleys, plains, and mid-to-high hills. High rates of unemployment have driven a surge of migration - particularly of younger people - to Kathmandu and abroad. Indrasarowar rural municipality, selected for this project, is located in the north-eastern part of the district in which the majority of the population belong to Tamang ethnic group (80%). Just over 9% of the population belong to the Newar ethnic group, who are the historical inhabitants of the Kathmandu Valley, while a further 9.6% of the population are Hill Brahmin/Chhetri. Just over 70% of the population speak Tamang, with 26.5% speaking Nepali.

Dhanusha is one of the southern Terai districts of Janakpur Zone and is an agriculture dependent district. Janakpurdham city is the capital of State 2 where this district is located and one of the commercial centres in the Terai region. It is also the administrative hub of the state. Laxminiya rural municpality is located around 5 km from the major city of Janakpur; the settlement straddles a highway, allowing for some road access. The main livelihood is subsistence farming. The majority of people in this rural municipality belong to Madhesi middle caste (i.e. Yadav, Sudhi, Lohar, Sonar; this is a new term recently accepted into official vocabulary to describe this separate group in the Nepali social classification system) and Maithili is their main language. This is the most densely populated area included in the study.

Initial discussions with the key stakeholders included 15 Key Informant Interviews (KIIs) and 19 Focus Group Discussions (FGDs) with Female Community Health Volunteers (FCHV), teachers, social workers and health care staff. This important first stage of the study, which took place over a six-month period in the first half of 2018, allowed the researchers to develop their understanding of the general context of the burn injuries. Participant observation sessions (n=13) that focused on food storage and cooking practices, together with social maps and seasonal calendars, were used to augment the narrative on burn injuries, their prevention and management in each of the rural municipalities. Based on this initial work, items for the survey were prepared both in Nepali and English and reviewed by 5 local experts (including Sagun board members and university staff) to

For peer review only

enhance their validity. The data collection tool asked households to report on any burn injury that required medical attention (which could include a medical post, a health worker, a Female Community Health Volunteer, or a pharmacy) in the previous 12 months.

Survey data was collected in July and August 2018 using a structured questionnaire to collect data on social demographic and household descriptors; incidences of burn injury meeting the case definition first aid received and health seeking behaviour after the burn injury. The questionnaire was pre-tested in Mangaltar of Kavre district, where it was piloted on 15 households; only minor changes were required at the end of this phase. The final version of the survey tool was developed into an electronic format (CSPro software) in order to assist with the logistics of collecting data in remote areas, where smart phones are used routinely despite the geographic remoteness.

All enumerators, from a pool of experienced individuals regularly used by Sagun, were trained in data collection using an electronic version of the form on smart phones to ensure consistency of use and reduce human error. The training included a day on theory and a day on practical issues, followed up by 3 supervised community-based training opportunities resulting in 3-5 full days of supervised training. All enumerators were instructed to call at the selected households up to 3 times until someone was present to complete the questionnaire: after 3 attempts no further attempt was made to contact the household members.

Three introductory visits were organised in each RM to discuss the details of the research proposal with representatives of the local government, health care staff and other relevant stakeholders at the RM level. A letter of approval was provided by each of the relevant local governments. The research proposal was reviewed and approved by Nepal Health Research Council (NHRC-Reg. no.348/2018), with written consent taken from each household. Survey data was recorded and analysed using IBM SPSS version 23. The unit of analysis was taken to be 'the household'.

The majority of the analysis was descriptive in nature and involved summarising findings by region, gender, type of burn, initial first aid and final destination of health service need. The data are presented for the whole cohort, as well as by region. The regional data are important to ensure the range of local experiences are reflected appropriately: the data for the whole cohort are important in order to provide policy makers with the relevant information for national strategies. Our experiences in the area have highlighted that the local communities feel that they are frequently being asked to participate in surveys – yet feel that nothing changes. So, although it is important to

For peer review only - http://bmjopen.bmj.com/site/about/guidelines.xhtml

fully understand the baseline situation so that we can evaluate whether our future interventions have been effective, we have also sought ways of presenting our findings to the local community that are easy to understand across languages. The focus has been to highlight the ways in which people access burn care by understanding their pathway from injury to treatment as a first step in co-creating prevention strategies that are locally relevant and implementable.

Patient and Public Involvement

The public and former patients were involved in the design of the study through interviews with key personnel, by allowing the researchers to observe their everyday practices (for example, routine cooking and food storage practices) and through community focus groups (n=19). The key stakeholders involved included political leaders, health post workers, household members (including burn survivors), women's groups, and schoolteachers. These same groups were involved in the direct dissemination of the community findings using info-grams and case-based narratives, and will help to develop locally relevant and locally owned awareness campaigns, prevention strategies and educational materials to support appropriate first-aid practices for the next phase of the study.

Results

1305 households were included in the original sampling frame; data were recorded from 1279 (98% of the initial sample). In the 12 months preceding the survey, 2.7% of the households reported at least one serious burn injury (see Table 1). Of the three RM's, Laxminiya reported 4 of every 100 households experiencing a burn injury, Indrasarowar reported 1:100, while Ama Chhodingmo reported 3:100. More women were affected, scalds being the most frequent cause of injury and the injury was most likely to happen at home; more injuries were reported in adults than children over the 12-month time frame.

Table 2 presents descriptive data on knowledge and practices on burn prevention, first aid and health seeking behaviour in all 1279 households. Exposure to prevention education was limited, with only one in ten households self-reported exposure to education or information about first aid treatment for burns. Two-thirds of the households indicated that they believe that most burns were preventable, with a significantly greater proportion (74%) in Laxminiya; interestingly, this is where the rates for burn injury in the last 12 months were the highest.

The most frequent response to first aid response was the use of water when a family member was scalded (27.4%), with the application of lotion as the lowest response (5.7%). However, Table 2 also

For peer review only - http://bmjopen.bmj.com/site/about/guidelines.xhtml

outlines regional variations with Laximiniya reporting the highest use of water (38.6%) whereas the highest response in Indrasarowar is to apply tomato, and the highest response in Ama Chhodingmo is to use a range of other options (including mango leaf paste, cow dung, mud, and herbs). Households in Ama Chhodingmo also reported that 28.2% say they would not know what to do in such circumstances.

Forty percent would take off clothing if they caught fire (40.4%); assuming it was available, 3 in 10 households reported they would either pour water onto the victim or jump into water. Only 3 in every 100 households reported that they would stop, drop and roll, while about 1 in 10 reported they would smother the flames with a cloth. One out of every 10 households indicated they would not know what to do. These overall figures again hide substantial differences in regional responses (Table 2).

The majority of Households responded that they would take a family member to hospital following a major burn (73.4%) or to a primary healthcare facility for a minor burn (57.5%). There were regional differences, with only a little over half of households in Ama Chhodingmo reporting they would take a family member to hospital immediately after a large or major burn (55.6%). One third of all households reported that they would manage minor burn incidents at home immediately after the incident, which was consistent across the three sites.

The data relating the experiences of those injured in the previous 12 months have been summarised in an info-gram (Figure 1) which illustrates patient journeys from initial injury to treatment; each identified case from the data base has been given an icon to reflect whether they are male or female, and an adult or a child. The left hand side of the icon reflects the type of burn injury, with the right hand side reflecting their rural municipality. For example, Case A is a female child from Laximinya, who experienced a flame burn, was treated initially with an alternative form of first aid (tube/lotion) and was transferred by ambulance 5 hours after injury and then spent 35 days in hospital. Ten reported cases did not seek any formal treatment beyond first aid; eleven cases reported to a primary care facility, one of which (B4) was referred onto secondary/tertiary care where she stayed for 7 days; the remaining 13 cases presented directly to secondary/tertiary care where the average length of hospital stay was 21 days.

Figure 1 shows that only 5 out of 34 burns received appropriate first aid in the form of cooling the wound prior to applying alternative methods for first aid. One in 4.4 burn victims applied some form

For peer review only - http://bmjopen.bmj.com/site/about/guidelines.xhtml

of tube based lotion or medicine as a first aid measure indicating the availability of some form of 'over the counter' burn remedy. Minor burns that can be managed at health posts or pharmacies were filtered appropriately by self-referral, while major or serious burns presented to secondary or tertiary hospitals. The data indicate that of the most serious burn cases requiring admission are disproportionately experienced by women/girls; in all cases they are linked to cooking activities. Figure 2, which summarises the economic and social costs for the 24 individuals who received formal treatment, shows that more than half reported having to borrow money or sell assets to cover the costs of their treatment. One in four suffered a disability that resulted in full dependence to perform one or more daily tasks.

Discussion

The average time spent at primary vs secondary or tertiary level in the patient journey suggests that many community members were able to appropriately self-select where they should present for medical treatment. However, responses from Ama Chhodingmo suggested that only a half of households would take serious cases directly to hospital, which may be a reflection of lack of access in terms of resources - financial and otherwise, or physical distance to secondary or tertiary level services.

Our data on self-report of serious burn injury, requiring secondary or tertiary care are substantially higher than we anticipated, based on the Annual Report[9], and reflect a substantial number of households that have experienced a serious burn, many of whom needed hospital care and will likely need on-going management for their pain, disabilities and limitations in function, and ability to be self-sufficient.

Together with the background information collected in the initial phases of the study, the survey provides great insight into the risk factors and prevailing knowledge of burn injury at the rural municipality level, as well as the personal financial costs borne as a consequence of burn injuries. Presenting this information in a simple visual format to local stakeholder groups will enable informed discussion and support the co-creation approach that will empower local communities to implement prevention activities.

Our ability to plot the experiences of individuals from initial injury through to length of hospital stay, will allow local health teams to work in tandem with local communities to fully explain the importance of initial first aid and the consequences of delayed (or inappropriate) treatment. The

For peer review only - http://bmjopen.bmj.com/site/about/guidelines.xhtml

use of info-grams, to support patient understanding of health messages[10], has enabled us to summarise the data in an easily digestible format and will allows us to work with a wide range of stakeholders. Although some translation will be required, the emphasis on imagery should support us to disseminate messages to health care professionals across the country. Initial work on scoping out a prevention strategy has already highlighted the importance of this local knowledge when interpreting the data, and of infograms as a focal point for meaningful discussions when co-creating a context-relevant prevention strategy.

These data suggest that the key areas for prevention would be cooking within the home setting, particularly for women and children. Local knowledge will be extremely important in identifying how this needs to be built into a program. First aid messages need to stress the importance of cool clean water as an initial treatment, when it is available (particularly for scald and flame burns); however, programs will need to reflect on what is locally available and differentiate between acceptable, alternative cooling options and those that must be avoided (e.g., mud). When clothing catches fire, household members need to be aware of the stop, drop and roll method and prioritise that no additional body parts (or other individuals or items) catch fire in the process.

This was a robustly designed study with a strong emphasis on using local knowledge to inform the content of the data collection tool, and substantial emphasis was placed on spending time with the local communities to ensure their full involvement. However, it is important not to over interpret the findings of the survey at either district or national level; any method that relies on recall is subject to memory bias. In this study, the proportional distribution of households recruited roughly reflected the relative population sizes of the RM's in each district.

Only one member of each household, even in households with multiple burn injuries, was interviewed about the incident; the individual selected was the one with the most serious injury meeting the case definition. This approach may have resulted in a potential underestimation of burn injuries and any new studies should consider interviewing all those with a serious burn injury.

The team involved in the initial stages of the project are already working, in collaboration with the research team, to co-create appropriate prevention strategies including a wide range of locally relevant activities, where the local community propose ideas that would make a real difference for their own communities. This is an important initial step in developing locally relevant and locally

For peer review only - http://bmjopen.bmj.com/site/about/guidelines.xhtml

owned prevention strategies to help reduce the incidence of burns, whilst ensuring that those with an injury receive the most appropriate first aid and seek appropriate care as quickly as possible.

Acknowledgements

We would especially like to acknowledge the support of Mr Bindeshwor Yadav, Chairperson, Laxminiya Rural Municipality, Dhanusha; Mr Buchung Tamang, Chairperson, Ama Chhodingmo Rural Municipality, Rasuwa; and Mr Jivan Lama, Chairperson, Indrasarowar Rural Municipality, Makawanpur.

With thanks to Arjun Bhattarai for his support in developing the online tool for data capture and support with the sampling frame.

The project team would like to express their heartfelt thanks to everyone who took the time to participate in the survey and to the local SAGUN team members who are so enthusiastic to bring about change and improvements in their local communities in Nepal.

Contribution of authors

Kamal Phuyal: Local lead for SAGUN in Nepal and the local research contact for the survey

Edna Ogada: Research assistant with responsibility for assisting with the ethical approval process, organising and analysing the data

Richard Bendell: Program manager for InterBurns, supported the logistics for the project and the design of the data collection tool

Patricia Price: Academic lead and responsible for writing the paper

Tom Potokar: Grant holder and Chief Investigator.

All authors have reviewed the paper prior to submission

Competing Interests

There are no competing interests for any of the authors.

For peer review only - http://bmjopen.bmj.com/site/about/guidelines.xhtml

Funding

This project is funded by the National Institute for Health Research (NIHR), NIHR Global Health Research Group on Burn Trauma, Grant Reference 16/137/110. The views expressed are those of the authors and not necessarily those of the NIHR of the Department of Health and Social Care.

Data Sharing

Data will be available from NIHR Open Data supplied by NIHR Centre for Business Intelligence, Crown Copyright and database licensed under the Open Government Licence (http://www.nationalarchives.gov.uk/doc/open-government-licence/version/3/): Global Health Research (in development). Data will also be archived at Swansea University.

References

1. Peck M, Molnar J & Swart D. A global plan for burn prevention and care. *Bulletin of the World Health Organisation* 2009;**87**: 802-803 doi: 10.2471/BLT.08.059733
2. WHO Factsheet on Burns: March 2018 (accessed May 7 2019) available at http://www.who.int/news-room/fact-sheets/detail/burns
3. Stokes, MAR & Johnson WD. Burns in the Third World: an unmet need. *Annals of Burns Fire Disasters*. 2017 Dec 31; **30**(4): 243–246.
4. Mathers CD, Bernard C, Moesgaard Iburg K, *et al*. 2003. Global Burden of Disease in 2002: data sources, methods and results (Version 2). *Global Programme on Evidence for Health Policy Discussion* Paper No.**54**. World Health Organisation; Geneva http://www.who.int/healthinfo/paper54.pdf
5. Sengoelge M, El-Khatib Z & Laflamme L. The global burden of child burn injuries in the light of country level economic development. *Preventive Medicine Reports* 2017; **6**: 115-120 http://dx.doi.org/10.1016/j.p.medr.2017.02.024
6. Public Health Institute. *Survey on risk factors for burn injuries in 0-5 year old children and related knowledge, attitudes and practices of their caregivers.* Ulaanbaatar: Ministry of Health of Mongolia 2009
7. Tripathee S & Basnet SJ Epidemiology of burn injuries in Nepal: a systematic review. *Burns and Trauma* 2017 **5**:10 doi 10.1186/s41038-017-0075-y
8. Ministry of Health and Population. *Annual Report,* 2016. Department of Health Services, Kathmandu, Nepal

For peer review only - http://bmjopen.bmj.com/site/about/guidelines.xhtml

9. Sethi D, Habibula S, McGee K, *et al*. (Eds) *Guidelines for Conducting Community Surveys on Injuries and Violence.* 2004 World Health Organisation, Geneva ISBN: 92 4 154 648 4

10. Barros IM, Alcantara TS, Mesquita M *et al* The use of pictograms in health care: a literature review. *Res Social Admi Pharm* 2014, Sept-Oct; **10** (5): 704-19 http://doi:10.1016/j.sapharm.2-13.11.002

For peer review only

Table 1: Prevalence for burn injury in three rural municipalities in Nepal, 2017- 2018 (n= 1279 Households)

**% responses**	Indrasarowar, Makwanpur (n=415)	Laxminiya, Dhanusha (n=630)	Ama Chhodingmo, Rasuwa (n=234)	**Total**	**95 % CI**
at least one burn injury	1.2	3.5	3.0	2.7	1.8,3.7
**who was burned**					
children	0.5	1.4	1.3	1.1	0.6, 1.8
Total adults	0.7	2.1	1.7	1.6	1.0,2.4
women	0.7	2.4	1.7	1.7	1.1, 2.6
men	0.5	1.1	1.3	0.9	0.5, 1.6
**cause of burn**					
flames	0.0	1.7	0.4	0.9	0.5,1.6
scalding	1.0	1.6	1.7	1.4	0.8,2.2
other mechanisms	0.2	0.2	0.9	0.3	0.1,0.8
**where the burn injury occurred**					
at home	1.2	3.5	2.1	2.5	1.7,3.5
away from the home	0.0	0.0	0.9	0.2	0.0,0.7
**location of injury**					
head and neck	0.0	0.5	0.0	0.2	0.0,0.7
limbs	1.0	3.0	2.1	2.2	1.5,3.1
trunk	0.2	0.0	0.9	0.2	0.0,0.7
**whether the injury occurred during cooking**					
while cooking	1.2	3.5	2.1	2.5	1.7,3.5
other activities	0.0	0.0	0.0	0.0	0.0

For peer review only

Table 2: Burn prevention, first aid and health seeking behaviour in Nepal, 2017- 2018

**% responses**	Indrasarowar, Makwanpur(n=415)	Laxminiya, Dhanusha(n=630)	Ama Chhodingmo, Rasuwa(n=234)	**Total**	**95 % CI**
**burn prevention**					
received education on prevention in past 12 months	13.5	10.8	6.8	10.9	9.3,12.8
believe most burns are preventable	52.3	73.8	47.4	62.0	59.3,64.7
believe most people with burns die as a result of their injury	68.0	82.2	70.1	75.4	72.9,77.7
believe that burns can cause bad scars	85.1	96.5	85.5	90.8	89.1,92.3
**first aid**					
received education on first aid in past 12 months	15.7	10.5	5.6	11.3	9.6,13.1
use water	18.3	38.6	13.2	27.4	24.9,29.9
tomato	39.3	5.9	14.1	18.2	16.1,20.4
potato paste	0.0	25.4	0.0	12.5	10.7,14.4
aloe vera	20.0	2.5	1.7	8.1	6.6, 9.7
honey	0.0	13.3	0.0	6.6	5.3,8.1
lotion/tube medicine	5.1	4.6	9.8	5.7	4.5,7.1
other (in order of frequency mango leaf paste, cow dung, mud, herbs, various oils and plants)	5.5	5.9	32.9	10.7	9.1,12.5
would not know what to do	11.8	3.8	28.2	10.9	9.2,12.7
**Clothing catching on fire**					
stop, drop and roll	1.7	5.9	0.0	3.4	2.5,4.6
smother flames with a cloth	13.0	16.7	3.4	13.1	11.3,15
jump in water	7.7	11.6	1.3	8.4	7,10.1
take off the clothing	28.4	53.8	25.6	40.4	37.7,43.2
pour water	34.7	8.7	31.6	21.3	19.1,23.7
would not know what to do	13.0	1.7	36.3	11.7	10, 13.6
**Health seeking behaviour (large or major burn)**					
hospital immediately after incident	67.7	83.8	55.6	73.4	70.9,75.8

For peer review only - http://bmjopen.bmj.com/site/about/guidelines.xhtml

% responses	Indrasarowar, Makwanpur(n=415)	Laxminiya, Dhanusha(n=630)	Ama Chhodingmo, Rasuwa(n=234)	Total	95 % CI
primary health care facility* immediately after incident	29.2	15.5	40.6	24.6	22.2,27.0
**Health seeking behaviour (minor burn)**					
manage at home	33.7	37.6	34.6	35.8	33.2, 38.5
primary health care facility*	58.3	58.6	53.0	57.5	54.7,60.2
hospital	1.7	2.4	5.6	2.7	(1.9, 3.8)

*Mainly health post but some pharmacy

Figure 1: Patient journeys, burn injuries in the previous 12 months

Legend: Each participant has been given a unique identifier, colour coded to show the type of injury and the rural municipality where the participant lived. The average, median and mode are given for 1) time of presentation at a health care facility since the injury took place, and 2) the length of stay at the location of treatment.

- to be inserted –

Figure 2: Economic costs and overall impact of burn injuries in the previous 12 months

Legend: Each participant has the same unique identifier as in Figure 1

- to be inserted –

For peer review only

For peer review only - http://bmjopen.bmj.com/site/about/guidelines.xhtml

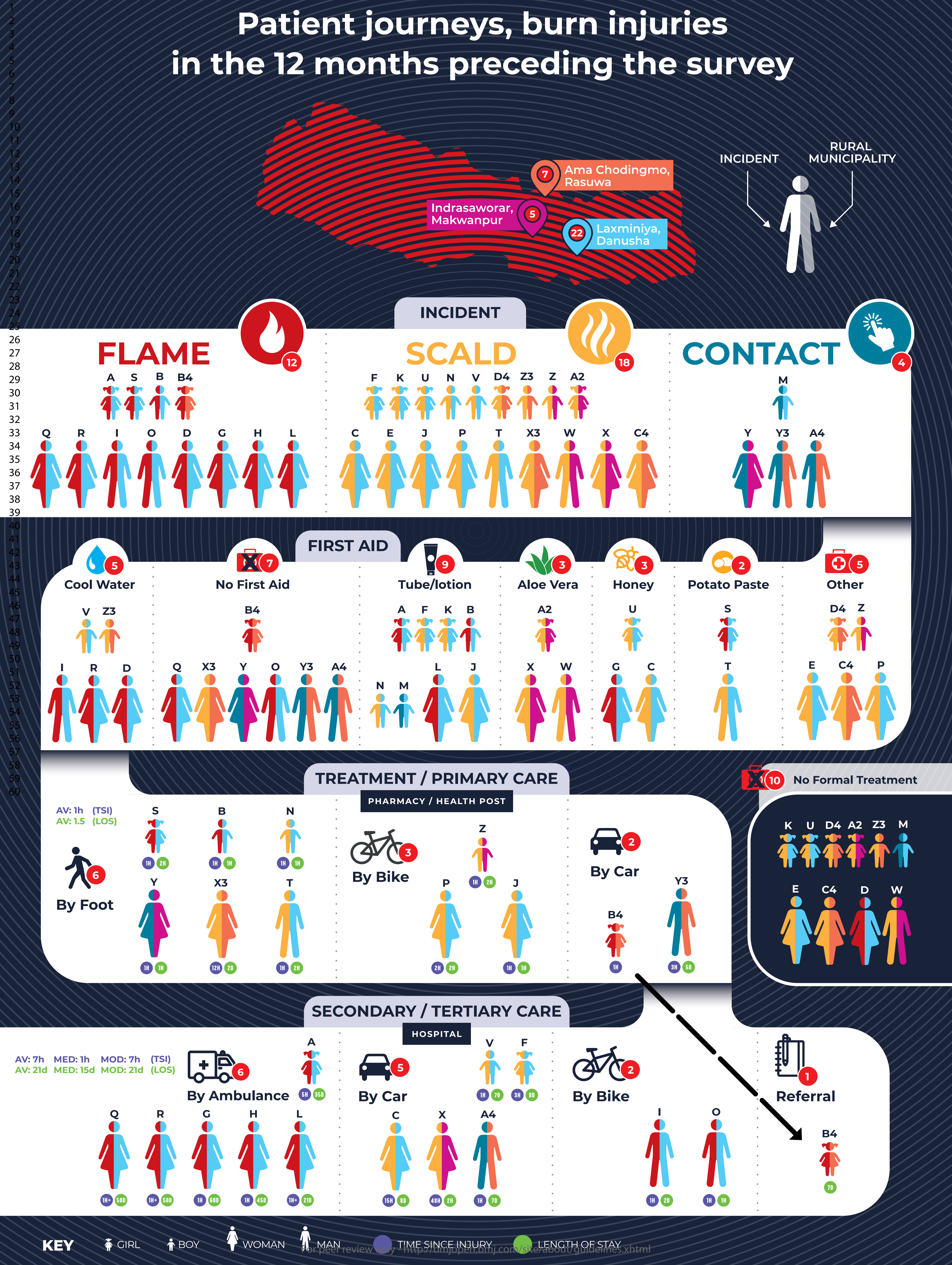
Patient journeys, burn injuries
in the 12 months preceding the survey
7 Ama Chodingmo, Rasuwa
5 Indrasaworar, Makwanpur
22 Laxminiya, Danusha
INCIDENT
RURAL MUNICIPALITY
INCIDENT
FLAME 12
A S B B4
Q R I O D G H L
SCALD 18
F K U N V D4 Z3 Z A2
C E J P T X3 W X C4
CONTACT 4
M
Y Y3 A4
FIRST AID
Cool Water 5
V Z3
I R D
No First Aid 7
B4
Q X3 Y O Y3 A4
Tube/lotion 9
A F K B
N M L J
Aloe Vera 3
A2
X W
Honey 3
U
G C
Potato Paste 2
S
T
Other 5
D4 Z
E C4 P
TREATMENT / PRIMARY CARE
PHARMACY / HEALTH POST
AV: 1h (TSI)
AV: 1.5 (LOS)
By Foot 6
S B N
Y X3 T
By Bike 3
Z
P J
By Car 2
B4 Y3
No Formal Treatment 10
K U D4 A2 Z3 M
E C4 D W
SECONDARY / TERTIARY CARE
HOSPITAL
AV: 7h MED: 1h MOD: 7h (TSI)
AV: 21d MED: 15d MOD: 21d (LOS)
By Ambulance 6
A
Q R G H L
By Car 5
V F
C X A4
By Bike 2
I O
Referral 1
B4
KEY
GIRL
BOY
WOMAN
MAN
TIME SINCE INJURY
LENGTH OF STAY

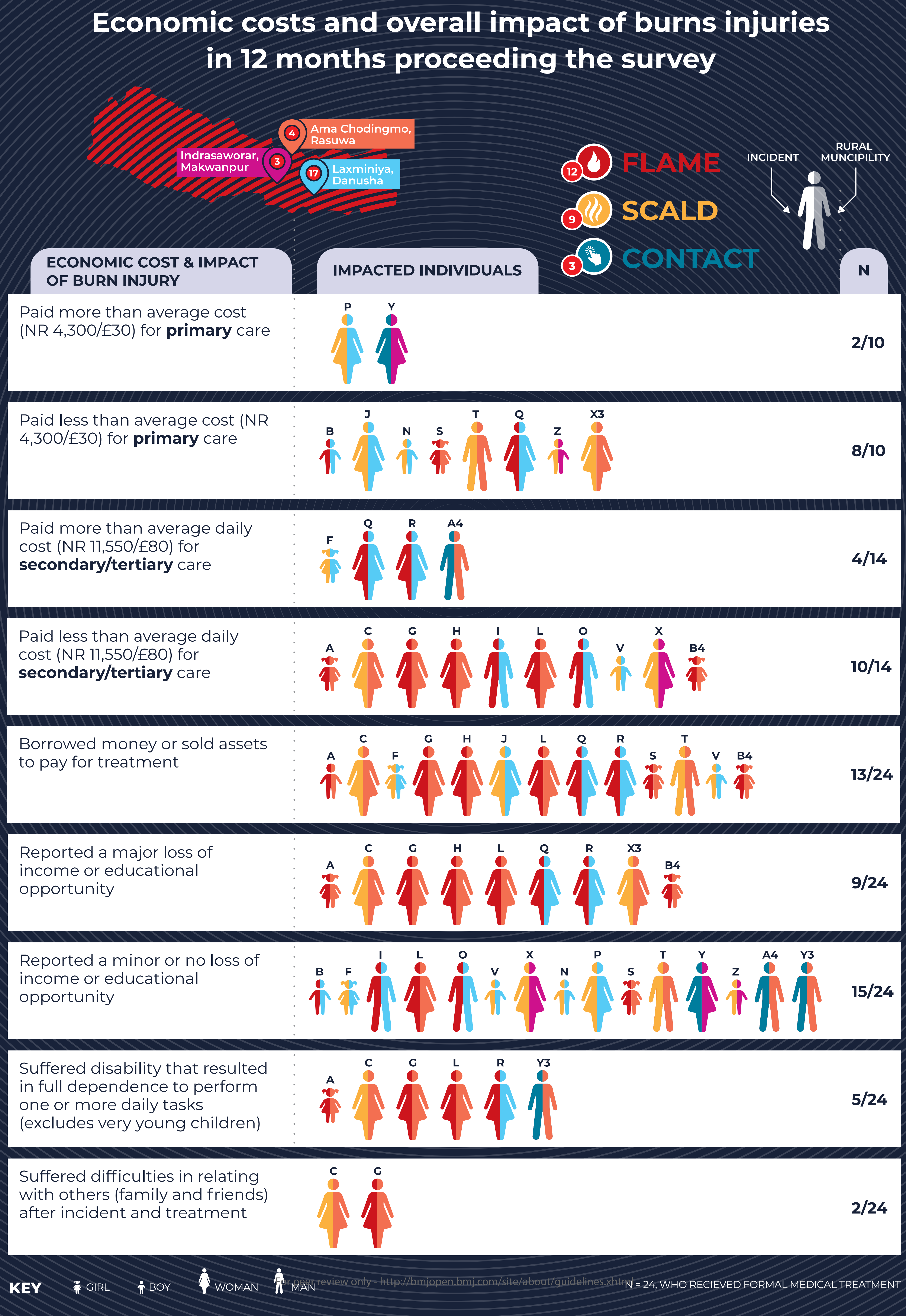

For peer review only - http://bmjopen.bmj.com/site/about/guidelines.xhtml

STROBE Statement—checklist of items that should be included in reports of observational studies

	Item No	Recommendation
**Title and abstract** **Page 1**	1	(*a*) Indicate the study's design with a commonly used term in the title or the abstract
		(*b*) Provide in the abstract an informative and balanced summary of what was done and what was found
**Introduction**		
Background/rationale Page 3	2	Explain the scientific background and rationale for the investigation being reported
Objectives Page 4	3	State specific objectives, including any prespecified hypotheses
**Methods**		
Study design Page 4	4	Present key elements of study design early in the paper
Setting Page 4; dates page 6	5	Describe the setting, locations, and relevant dates, including periods of recruitment, exposure, follow-up, and data collection
Participants Page 4-5	6	(*a*) *Cohort study*—Give the eligibility criteria, and the sources and methods of selection of participants. Describe methods of follow-up<br>*Case-control study*—Give the eligibility criteria, and the sources and methods of case ascertainment and control selection. Give the rationale for the choice of cases and controls<br>*Cross-sectional study*—Give the eligibility criteria, and the sources and methods of selection of participants
		(*b*) *Cohort study*—For matched studies, give matching criteria and number of exposed and unexposed<br>*Case-control study*—For matched studies, give matching criteria and the number of controls per case
Variables N/A	7	Clearly define all outcomes, exposures, predictors, potential confounders, and effect modifiers. Give diagnostic criteria, if applicable
Data sources/ measurement N/A	8*	For each variable of interest, give sources of data and details of methods of assessment (measurement). Describe comparability of assessment methods if there is more than one group
Bias N/A	9	Describe any efforts to address potential sources of bias
Study size Page 4	10	Explain how the study size was arrived at
Quantitative variables Page 6-7	11	Explain how quantitative variables were handled in the analyses. If applicable, describe which groupings were chosen and why
Statistical methods Page 6	12	(*a*) Describe all statistical methods, including those used to control for confounding
		(*b*) Describe any methods used to examine subgroups and interactions
		(*c*) Explain how missing data were addressed
		(*d*) *Cohort study*—If applicable, explain how loss to follow-up was addressed<br>*Case-control study*—If applicable, explain how matching of cases and controls was addressed<br>*Cross-sectional study*—If applicable, describe analytical methods taking account of sampling strategy
		(*e*) Describe any sensitivity analyses

For peer review only

For peer review only - http://bmjopen.bmj.com/site/about/guidelines.xhtml

**Results**		
Participants<br>Page 7	13*	(a) Report numbers of individuals at each stage of study—eg numbers potentially eligible, examined for eligibility, confirmed eligible, included in the study, completing follow-up, and analysed
		(b) Give reasons for non-participation at each stage
		(c) Consider use of a flow diagram
Descriptive data<br>Page 7	14*	(a) Give characteristics of study participants (eg demographic, clinical, social) and information on exposures and potential confounders
		(b) Indicate number of participants with missing data for each variable of interest
		(c) *Cohort study*—Summarise follow-up time (eg, average and total amount)
Outcome data<br>N/A	15*	*Cohort study*—Report numbers of outcome events or summary measures over time
		Case-control study—Report numbers in each exposure category, or summary measures of exposure
		Cross-sectional study—Report numbers of outcome events or summary measures
Main results<br>N/A	16	(*a*) Give unadjusted estimates and, if applicable, confounder-adjusted estimates and their precision (eg, 95% confidence interval). Make clear which confounders were adjusted for and why they were included
		(*b*) Report category boundaries when continuous variables were categorized
		(*c*) If relevant, consider translating estimates of relative risk into absolute risk for a meaningful time period
Other analyses<br>N/A	17	Report other analyses done—eg analyses of subgroups and interactions, and sensitivity analyses
**Discussion**		
Key results<br>Page 9	18	Summarise key results with reference to study objectives
Limitations<br>Page 10	19	Discuss limitations of the study, taking into account sources of potential bias or imprecision. Discuss both direction and magnitude of any potential bias
Interpretation<br>Page 10	20	Give a cautious overall interpretation of results considering objectives, limitations, multiplicity of analyses, results from similar studies, and other relevant evidence
Generalisability<br>Page 10	21	Discuss the generalisability (external validity) of the study results
**Other information**		
Funding<br>Page 11	22	Give the source of funding and the role of the funders for the present study and, if applicable, for the original study on which the present article is based

*Give information separately for cases and controls in case-control studies and, if applicable, for exposed and unexposed groups in cohort and cross-sectional studies.

Note: An Explanation and Elaboration article discusses each checklist item and gives methodological background and published examples of transparent reporting. The STROBE checklist is best used in conjunction with this article (freely available on the Web sites of PLoS Medicine at http://www.plosmedicine.org/, Annals of Internal Medicine at http://www.annals.org/, and Epidemiology at http://www.epidem.com/). Information on the STROBE Initiative is available at www.strobe-statement.org.

For peer review only

BMJ Open

Burns in Nepal: a participatory, community survey of burn cases and knowledge, attitudes and practices to burn care and prevention in 3 rural municipalities

Journal:	*BMJ Open*
Manuscript ID	bmjopen-2019-033071.R2
Article Type:	Original research
Date Submitted by the Author:	19-Dec-2019
Complete List of Authors:	Phuyal, Kamal; SAGUN: a search for harmony<br>Ogada, Edna; Swansea University, Centre for Global Burn Injury Policy and Research, College of Human and Health Sciences<br>Bendell, Richard; Interburns<br>Price, Patricia; Swansea University; Interburns<br>Potokar, Tom; Swansea University, Centre for Global Burn Injury, Policy and Research, College of Human and Health Sciences; InterBurns
**Primary Subject Heading**:	Public health
Secondary Subject Heading:	Epidemiology
Keywords:	Burns, Prevention, Community

SCHOLARONE™
Manuscripts

I, the Submitting Author has the right to grant and does grant on behalf of all authors of the Work (as defined in the below author licence), an exclusive licence and/or a non-exclusive licence for contributions from authors who are: i) UK Crown employees; ii) where BMJ has agreed a CC-BY licence shall apply, and/or iii) in accordance with the terms applicable for US Federal Government officers or employees acting as part of their official duties; on a worldwide, perpetual, irrevocable, royalty-free basis to BMJ Publishing Group Ltd ("BMJ") its licensees and where the relevant Journal is co-owned by BMJ to the co-owners of the Journal, to publish the Work in this journal and any other BMJ products and to exploit all rights, as set out in our licence.

The Submitting Author accepts and understands that any supply made under these terms is made by BMJ to the Submitting Author unless you are acting as an employee on behalf of your employer or a postgraduate student of an affiliated institution which is paying any applicable article publishing charge ("APC") for Open Access articles. Where the Submitting Author wishes to make the Work available on an Open Access basis (and intends to pay the relevant APC), the terms of reuse of such Open Access shall be governed by a Creative Commons licence – details of these licences and which Creative Commons licence will apply to this Work are set out in our licence referred to above.

Other than as permitted in any relevant BMJ Author's Self Archiving Policies, I confirm this Work has not been accepted for publication elsewhere, is not being considered for publication elsewhere and does not duplicate material already published. I confirm all authors consent to publication of this Work and authorise the granting of this licence.

For peer review only

Title: *Burns in Nepal: a participatory, community survey of burn cases and knowledge, attitudes and practices to burn care and prevention in 3 rural municipalities*

Authors:

Kamal Phuyal[1], Edna Ogada[2], Richard Bendell[3], Patricia Price [2,3], Tom Potokar [2,3]

1. SAGUN: a search for harmony; Kupandol, Lalitpur, Nepal
2. Centre for Global Burn injury Policy and Research, Swansea University
3. InterBurns, Welsh Centre for Burns and Plastic Surgery, Swansea

Corresponding author:

Professor Patricia Price,

Centre for Global Burn injury Policy and Research,

College of Human and Health Sciences

Room 26 Haldane Building

Swansea University

Singleton Park

Swansea SA2 8PP

Email: p.e.price@swansea.ac.uk

Keywords:

Burns/epidemiology: Burns/prevention: Community participation: Survey: First Aid

For peer review only

Abstract

Objectives: As part of an on-going, long-term project to co-create burn prevention strategies in Nepal, we collected baseline data to share and discuss with the local community, use as a basis for a co-created prevention strategy and then monitor changes over time. This paper reports on the method and outcomes of the baseline survey and demonstrates how the data are presented back to the community.

Design: a community-based survey

Setting: Community based in 3 Rural Municipalities in Nepal.

Participants: 1305 households were approached: the head of 1279 households participated, giving a response rate of 98%. In 90.3% of cases the head of the household was male.

Results

We found that 2.7% (CI: 1.8, 3.7) of 1279 households, from 3 representative municipalities, reported at least one serious burn in the previous 12 months: a serious burn was defined as one requiring medical attention and/or inability to work or do normal activities for 24 hours. Whilst only 4 paediatric and 10 adult cases in the previous 12 months reached hospital care, the impact on the lives of those involved was profound. Only one patient was referred on from primary to secondary/tertiary care; the average length of hospital stay for those presenting directly to secondary/tertiary care was 21 days. A range of first aid behaviours were used, many of which are appropriate for the local context whilst a few may be potentially harmful (e.g., the use of dung).

Conclusion

The participatory approach used in the study ensured a high response rate. We have demonstrated that infographics can link the pathway for each of the cases observed from initial incident to final location of care.

Article Summary:

Strengths

- Detailed and sustained work with the local community to ensure enhanced community participation in the survey
- Tracking individual patient journeys from injury to final destination for burn care
- The use of info-graphics to overcome language and knowledge barriers when presenting the findings back to the community.

Limitations

- Only one person with a burn injury per household was included in the study.

For peer review only

Introduction

The World Health Organisation has repeatedly tried to raise awareness of the fact that the majority of the burden of burn injury, from a global perspective, is borne by low and middle-income countries [1,2]. Other recent publications[3] have confirmed that the greater burden of mortality and morbidity related to burn injury is borne by the world's poor, residing mostly in LMICs. Patients in these countries continue to endure an extremely high level of suffering, with the rate of child deaths from burns being over 7 times higher in lower resource settings than in the western environment[4,5]. Mongolia has a particularly high rate of child burn injury as identified by The Global Burden of Disease study in 2013[5]; detailed community studies, conducted in Ulaanbaatar, have supported this finding and shown that 27% (N=247/900) had a history of burn injury[6]. A recent systematic review[7] found eight epidemiological studies based in Nepal and concluded that burn injuries are the leading cause of morbidity and mortality in the country. The study authors report mortality rates from 4.5% to 23.5% and conclude that the limited data explains the lack of effective intervention programmes in the area.

The Annual Report from the Department of Health and Population for Nepal, covering the fiscal year 2016-17, recorded 55,090 burn or scald injuries nationally[8]. Population statistics for the same time period were 28.9M, giving an incidence rate of 0.19% of patients with an injury sufficiently severe to seek help from an outpatient facility where data were recorded. Data from public hospitals (92% response rate) and private facilities (47.2% response rate) submitted as part of the Annual Report[8] and using the ICD codes T200 – T301 for inpatients, 1710 cases were reported with 1006 related to those below the age of 19, and 586 under the age of 5 (81.7% of these were aged 1-4). In the same time period there were 46 in-hospital deaths reported (31 F, 15 M) – although this does not include deaths from catastrophic events or from those who were sent straight to the mortuary.

Gupta et al[9] conducted a household survey using a cluster randomised cross section country wide survey in 15 districts in Nepal and reported a higher incidence of 55 burns from 1350 households (2%, 95% CI 1.5-2.6%). The majority of cases (60.4%) were due to hot liquid and/or hot objects. The study authors conclude that the demographics and aetiology of burns at a population level vary significantly from hospital level data. Tripathee and Basner[7], in their systematic review, stress the importance of epidemiological data as a pre-requisite for planning and implementing prevention programmes at the community level.

For peer review only - http://bmjopen.bmj.com/site/about/guidelines.xhtml

To bring about lasting changes in resource poor countries, it is vital that we have a detailed understanding of the current, local context including epidemiology, the availability of educational/prevention programmes and the ability of people in rural/remote areas to access vital first aid treatment in order to plan appropriate interventions. This paper focuses on the first part of a major initiative to address these questions and bring about change using an Implementation Science approach, based in Nepal. Whilst previous studies start to give us an indication of the scale of the problem, they do not give us information about the ways in which people sought help, what they did immediately after the injury, what local knowledge exists to provide first-aid and what prevention programmes are available locally. In order to ensure that we could fully evaluate our intervention, we needed to conduct a more in-depth community based survey so that we could develop and co-create (with community stakeholders) a community prevention and first aid strategy. This paper presents the findings of the baseline phase of the study. The specific objectives, related to selected areas of Nepal, were to: –

i. record the incidence and causes of burn injury in 2017-18,

ii. explore the knowledge, attitudes and practices of the community regarding burn care and prevention.

Methods

The on-going study uses a participatory action research design to develop a prevention and first aid strategy at the rural municipality level (RM) (n= 460 RM in Nepal). This first phase included a community sensitization and preparation plan, followed by baseline data collection. As the prevention strategies need to be developed at the rural municipality level, baseline data was collected in one randomly selected municipality in each of three districts, Ama Chhodingmo Rural Municipality (ARM) in Rasuwa, Indrasarowar Rural Municipality (IRM) in Makawanpur and Laxminiya Rural Municipality (LRM) in Dhanusha. These districts were drawn from three traditionally noted geographic regions of Nepal, Terai (flatland), Hills, and Himalayan regions. This purposive selection was used to ensure a degree of diversity in the communities studied, whilst representing the geo-political areas that any national policy would need to address.

The study population was all the people living in those rural municipalities, regardless of socio-demographic factors. A sample of 1305 households was selected from a population of 47,642, based on the WHO formula[10], adjusted to account for the relative population size in each district. Household selection was completed using a systematic sampling approach drawing from a listing of

For peer review only - http://bmjopen.bmj.com/site/about/guidelines.xhtml

the total number of households in each RM, formulated during the sensitisation preparation phase at each municipality office, when detailed discussions took place with local stakeholders.

Rasuwa district is located north of Kathmandu. It is a district of natural resources, particularly Langtang and Gosaikunda regions, which are well known for their natural beauty and trekking routes, with a high potential for eco-tourism businesses. However, poor roads, poor access to university education and poverty have hindered the development of the district. Ama Chhodingmo is located in the mountain region, bordering China to the north, and contains both natural and cultural diversity. The landscape in this area makes road construction very difficult, and most of the northern areas lack access to vehicle-friendly roads. Rasuwa itself is accessible by bus from Kathmandu via the Pasang Lhamu highway, but the administrative headquarters (Dhunche) are about 120 km from the capital. Over 98% of the population are Hill Ethnic Groups or Tamang; this is one of the indigenous inhabitant groups of Nepal, with their own distinct language, living in scattered hill villages.

Makawanpur is a hilly district, located on the southern border of Kathmandu. Despite bordering Kathmandu valley, many areas of Makwanpur remain inaccessible by road. The main livelihood for the majority of the population is subsistence farming. The district includes within its borders various agro-climatic zones: valleys, plains, and mid-to-high hills. High rates of unemployment have driven a surge of migration - particularly of younger people - to Kathmandu and abroad. Indrasarowar rural municipality, selected for this project, is located in the north-eastern part of the district in which the majority of the population belong to Tamang ethnic group (80%). Just over 9% of the population belong to the Newar ethnic group, who are the historical inhabitants of the Kathmandu Valley, while a further 9.6% of the population are Hill Brahmin/Chhetri. Just over 70% of the population speak Tamang, with 26.5% speaking Nepali.

Dhanusha is one of the southern Terai districts of Janakpur Zone and is an agriculture dependent district. Janakpurdham city is the capital of State 2 where this district is located and one of the commercial centres in the Terai region. It is also the administrative hub of the state. Laxminiya rural municipality is located around 5 km from the major city of Janakpur; the settlement straddles a highway, allowing for some road access. The main livelihood is subsistence farming. The majority of people in this rural municipality belong to Madhesi middle caste (i.e. Yadav, Sudhi, Lohar, Sonar; this is a new term recently accepted into official vocabulary to describe this separate group in the Nepali social classification system) and Maithili is their main language. This is the most densely populated area included in the study.

For peer review only - http://bmjopen.bmj.com/site/about/guidelines.xhtml

For peer review only

Initial discussions with the key stakeholders included 15 Key Informant Interviews (KIIs) and 19 Focus Group Discussions (FGDs) with Female Community Health Volunteers (FCHV), teachers, social workers and health care staff. This important first stage of the study, which took place over a six-month period in the first half of 2018, allowed the researchers to develop their understanding of the general context of the burn injuries. Participant observation sessions (n=13) that focused on food storage and cooking practices, together with social maps and seasonal calendars, were used to augment the narrative on burn injuries, their prevention and management in each of the rural municipalities. Based on this initial work, items for the survey were prepared both in Nepali and English and reviewed by 5 local experts (including Sagun board members and university staff) to enhance their validity. The data collection tool asked households to report on any burn injury that required medical attention (which could include a medical post, a health worker, a Female Community Health Volunteer, or a pharmacy) in the previous 12 months.

Survey data was collected in July and August 2018 using a structured questionnaire to collect data on social demographic and household descriptors, incidences of burn injury meeting the case definition, first aid received and health seeking behaviour after the burn injury. The questionnaire was pre-tested in Mangaltar of Kavre district, where it was piloted on 15 households; only minor changes were required at the end of this phase. The final version of the survey tool was developed into an electronic format (CSPro software) in order to assist with the logistics of collecting data in remote areas, where smart phones are used routinely despite the geographic remoteness.

All enumerators, from a pool of experienced participatory research data collectors regularly used by Sagun, were trained in data collection using an electronic version of the form on smart phones to ensure consistency of use and reduce human error. The training included a day on theory and a day on practical issues, followed up by 3 supervised community-based training opportunities resulting in 3-5 full days of supervised training. All enumerators were instructed to call at the selected households up to 3 times until someone was present to complete the questionnaire: after 3 attempts no further attempt was made to contact the household members.

Three introductory visits were organised in each RM to discuss the details of the research proposal with representatives of the local government, health care staff and other relevant stakeholders at the RM level. A letter of approval was provided by each of the relevant local governments. The research proposal was reviewed and approved by Nepal Health Research Council (NHRC-Reg.

For peer review only - http://bmjopen.bmj.com/site/about/guidelines.xhtml

no.348/2018), with written consent taken from each household. Survey data was recorded and analysed using IBM SPSS version 23. The unit of analysis was taken to be 'the household'.

The majority of the analysis was descriptive in nature and involved summarising findings by region, gender, type of burn, initial first aid and final destination of health service need. The data are presented for the whole cohort, as well as by region. The regional data are important to ensure the range of local experiences are reflected appropriately: the data for the whole cohort are important in order to provide policy makers with the relevant information for national strategies. Our experiences in the area have highlighted that the local communities feel that they are frequently being asked to participate in surveys – yet feel that nothing changes. So, although it is important to fully understand the baseline situation so that we can evaluate whether our future interventions have been effective, we have also sought ways of presenting our findings to the local community that are easy to understand across languages. The focus has been to highlight the ways in which people access burn care by understanding their pathway from injury to treatment as a first step in co-creating prevention strategies that are locally relevant and implementable.

Patient and Public Involvement

The public and former patients were involved in the design of the study through interviews with key personnel, by allowing the researchers to observe their everyday practices (for example, routine cooking and food storage practices) and through community focus groups (n=19). The key stakeholders involved included political leaders, health post workers, household members (including burn survivors), women's groups, and schoolteachers. These same groups were involved in the direct dissemination of the community findings using info-grams and case-based narratives, and will help to develop locally relevant and locally owned awareness campaigns, prevention strategies and educational materials to support appropriate first-aid practices for the next phase of the study.

Results

1305 households were included in the original sampling frame; data were recorded from 1279 (98% of the initial sample). In the 12 months preceding the survey, 2.7% of the households reported at least one serious burn injury (see Table 1). Of the three RM's, Laxminiya reported 4 of every 100 households experienced a burn injury, Indrasarowar reported 1:100, while Ama Chhodingmo reported 3:100. More women were affected, scalds being the most frequent cause of injury and the injury was most likely to happen at home; more injuries were reported in adults than children over the 12-month time frame.

For peer review only - http://bmjopen.bmj.com/site/about/guidelines.xhtml

Table 2 presents descriptive data on knowledge and practices on burn prevention, first aid and health seeking behaviour in all 1279 households. Exposure to prevention education was limited, with only one in ten households self-reported exposure to education or information about first aid treatment for burns. Two-thirds of the households indicated that they believe that most burns were preventable, with a significantly greater proportion (74%) in Laxminiya; interestingly, this is where the rates for burn injury in the last 12 months were the highest.

The most frequent response to first aid response was the use of water when a family member was scalded (27.4%), with the application of lotion as the lowest response (5.7%). However, Table 2 also outlines regional variations with Laximiniya reporting the highest use of water (38.6%) whereas the highest response in Indrasarowar was to apply tomato, and the highest response in Ama Chhodingmo was to use a range of other options (including mango leaf paste, cow dung, mud, and herbs). Households in Ama Chhodingmo also reported that 28.2% say they would not know what to do in such circumstances.

Forty percent would take off clothing if they caught fire (40.4%); 3 in 10 households reported they would either pour water onto the victim or jump into water. Only 3 in every 100 households reported that they would stop, drop and roll, while about 1 in 10 reported they would smother the flames with a cloth. One out of every 10 households indicated they would not know what to do. These overall figures again hide substantial differences in regional responses (Table 2).

The majority of Households responded that they would take a family member to hospital following a major burn (73.4%) or to a primary healthcare facility for a minor burn (57.5%). There were regional differences, with only a little over half of households in Ama Chhodingmo reporting they would take a family member to hospital immediately after a large or major burn (55.6%). One third of all households reported that they would manage minor burn incidents at home immediately after the incident, which was consistently reported across the three study sites.

The data relating the experiences of those injured in the previous 12 months have been summarised in an info-gram (Figure 1) which illustrates patient journeys from initial injury to treatment; each identified case from the data base has been given an icon to reflect whether they are male or female, and an adult or a child. The left hand side of the icon reflects the type of burn injury, with the right hand side reflecting their rural municipality. For example, Case A is a female child from

For peer review only - http://bmjopen.bmj.com/site/about/guidelines.xhtml

Laximinya, who experienced a flame burn, was treated initially with an alternative form of first aid (tube/lotion) and was transferred by ambulance 5 hours after injury and then spent 35 days in hospital. Ten reported cases did not seek any formal treatment beyond first aid; eleven cases reported to a primary care facility, one of which (B4) was referred onto secondary/tertiary care where she stayed for 7 days; the remaining 13 cases presented directly to secondary/tertiary care where the average length of hospital stay was 21 days.

Figure 1 shows that only 5 out of 34 burns received appropriate first aid in the form of cooling the wound prior to applying alternative methods for first aid. One in 4.4 burn victims applied some form of tube based lotion or medicine as a first aid measure indicating the availability of some form of 'over the counter' burn remedy. Minor burns that can be managed at health posts or pharmacies were filtered appropriately by self-referral, while major or serious burns presented to secondary or tertiary hospitals. The data indicate that of the most serious burn cases requiring admission are disproportionately experienced by women/girls; in all cases they are linked to cooking activities. Figure 2, which summarises the economic and social costs for the 24 individuals who received formal treatment, shows that more than half reported having to borrow money or sell assets to cover the costs of their treatment. One in four suffered a disability that resulted in full dependence to perform one or more daily tasks.

Discussion

Our data on self-report of serious burn injury, requiring secondary or tertiary care are substantially higher than we anticipated, compared to the Annual Report[8], and reflect a substantial number of households that have experienced a serious burn, many of whom needed hospital care and will likely need on-going management for their pain, disabilities and limitations in function, and ability to be self-sufficient. However, responses from Ama Chhodingmo suggested that only a half of households would take serious cases directly to hospital, which may be a reflection of lack of access in terms of resources - financial and otherwise, or physical distance to secondary or tertiary level services.

Together with the background information collected in the initial phases of the study, the survey provides great insight into the risk factors and prevailing knowledge of burn injury at the rural municipality level, as well as the personal financial costs borne as a consequence of burn injuries. Presenting this information in a simple visual format to local stakeholder groups will enable informed discussion and support the co-creation approach that will empower local communities to implement prevention activities.

For peer review only - http://bmjopen.bmj.com/site/about/guidelines.xhtml

Our ability to plot the experiences of individuals from initial injury through to length of hospital stay, will allow local health teams to work in tandem with local communities to fully explain the importance of initial first aid and the consequences of delayed (or inappropriate) treatment. The use of info-grams, to support patient understanding of health messages[11], has enabled us to summarise the data in an easily digestible format and will allows us to work with a wide range of stakeholders. Although some translation will be required, the emphasis on imagery should support us to disseminate messages to health care professionals across the country. Initial work on scoping out a prevention strategy has already highlighted the importance of this local knowledge when interpreting the data, and of infograms as a focal point for meaningful discussions when co-creating a context-relevant prevention strategy.

Our data suggest that the key areas for prevention would be cooking within the home setting, particularly for women and children. These data are supported by previous studies included in a systematic review[7]; these authors reported that 5 out of the 8 studies reviewed found more burns in females than males, and the most common location for a burn to occur was inside the house, mostly in the kitchen. One study included in the review reported that 86% of childhood burns occurred in the house. Local knowledge will be extremely important in identifying how this information needs to be built into a program. First aid messages need to stress the importance of cool clean water as an initial treatment, when it is available (particularly for scald and flame burns); however, programs will need to reflect on what is locally available and differentiate between acceptable, alternative cooling options and those that must be avoided (e.g., mud). When clothing catches fire, household members need to be aware of the stop, drop and roll method and prioritise that no additional body parts (or other individuals or items) catch fire in the process.

This was a robustly designed study with a strong emphasis on using local knowledge to inform the content of the data collection tool, and substantial emphasis was placed on spending time with the local communities to ensure their full involvement. However, it is important not to over interpret the findings of the survey at either district or national level; any method that relies on recall is subject to memory bias that can lead to inaccuracies in how the original incident was reported in the survey. In this study, the proportional distribution of households recruited roughly reflected the relative population sizes of the RM's in each district. Only one member of each household, even in households with multiple burn injuries, was interviewed about the incident; the individual selected was the one with the most serious injury meeting the case definition. Given that many households

For peer review only

in Nepal are multi-generational and large in size, the person interviewed may not have been aware of all burn incidents within the household so the possibility exists that on some occasions there may have been a more severe incident within the household that was not captured in this data set. This approach may have resulted in a potential underestimation of burn injuries and any new studies should consider interviewing all those with a serious burn injury.

The team involved in the initial stages of the project are already working, in collaboration with the research team, to co-create appropriate prevention strategies including a wide range of locally relevant activities, where the local community propose ideas that would make a real difference for their own communities. This is an important initial step in developing locally relevant and locally owned prevention strategies to help reduce the incidence of burns, whilst ensuring that those with an injury receive the most appropriate first aid and seek appropriate care as quickly as possible.

Acknowledgements

We would especially like to acknowledge the support of Mr Bindeshwor Yadav, Chairperson, Laxminiya Rural Municipality, Dhanusha; Mr Buchung Tamang, Chairperson, Ama Chhodingmo Rural Municipality, Rasuwa; and Mr Jivan Lama, Chairperson, Indrasarowar Rural Municipality, Makawanpur.

With thanks to Arjun Bhattarai for his support in developing the online tool for data capture and support with the sampling frame.

The project team would like to express their heartfelt thanks to everyone who took the time to participate in the survey and to the local SAGUN team members who are so enthusiastic to bring about change and improvements in their local communities in Nepal.

Contribution of authors

Kamal Phuyal: Local lead for SAGUN in Nepal and the local research contact for the survey

Edna Ogada: Research assistant with responsibility for assisting with the ethical approval process, organising and analysing the data

Richard Bendell: Program manager for InterBurns, supported the logistics for the project and the design of the data collection tool

For peer review only - http://bmjopen.bmj.com/site/about/guidelines.xhtml

Patricia Price: Academic lead and responsible for writing the paper

Tom Potokar: Grant holder and Chief Investigator.

All authors have reviewed the paper prior to submission

Competing Interests

There are no competing interests for any of the authors.

Funding

This project is funded by the National Institute for Health Research (NIHR), NIHR Global Health Research Group on Burn Trauma, Grant Reference 16/137/110. The views expressed are those of the authors and not necessarily those of the NIHR of the Department of Health and Social Care.

Data Sharing

Data will be available from NIHR Open Data supplied by NIHR Centre for Business Intelligence, Crown Copyright and database licensed under the Open Government Licence (http://www.nationalarchives.gov.uk/doc/open-government-licence/version/3/): Global Health Research (in development). Data will also be archived at Swansea University.

References

1. Peck M, Molnar J & Swart D. A global plan for burn prevention and care. *Bulletin of the World Health Organisation* 2009;**87**: 802-803 doi: 10.2471/BLT.08.059733
2. WHO Factsheet on Burns: March 2018 (accessed May 7 2019) available at http://www.who.int/news-room/fact-sheets/detail/burns
3. Stokes, MAR & Johnson WD. Burns in the Third World: an unmet need. *Annals of Burns Fire Disasters*. 2017 Dec 31; **30**(4): 243–246.
4. Mathers CD, Bernard C, Moesgaard Iburg K, *et al*. 2003. Global Burden of Disease in 2002: data sources, methods and results (Version 2). *Global Programme on Evidence for Health Policy Discussion* Paper No.**54**. World Health Organisation; Geneva http://www.who.int/healthinfo/paper54.pdf
5. Sengoelge M, El-Khatib Z & Laflamme L. The global burden of child burn injuries in the light of country level economic development. *Preventive Medicine Reports* 2017; **6**: 115-120 http://dx.doi.org/10.1016/j.p.medr.2017.02.024

For peer review only - http://bmjopen.bmj.com/site/about/guidelines.xhtml

6. Public Health Institute. *Survey on risk factors for burn injuries in 0-5 year old children and related knowledge, attitudes and practices of their caregivers.* Ulaanbaatar: Ministry of Health of Mongolia 2009

7. Tripathee S & Basnet SJ Epidemiology of burn injuries in Nepal: a systematic review. *Burns and Trauma* 2017 **5**:10 doi 10.1186/s41038-017-0075-y

8. Ministry of Health and Population. *Annual Report,* 2016. Department of Health Services, Kathmandu, Nepal

9. Gupta S, Mahmood U, Gurung S, et al. Burns in Nepal: A population based national assessment. *Burns* 2015 **41**(5): 1126-1132 doi:10.1016/j.burns.2014.11.012

10. Sethi D, Habibula S, McGee K, *et al*. (Eds) *Guidelines for Conducting Community Surveys on Injuries and Violence.* 2004 World Health Organisation, Geneva ISBN: 92 4 154 648 4

11. Barros IM, Alcantara TS, Mesquita M *et al* The use of pictograms in health care: a literature review. *Res Social Admi Pharm* 2014, Sept-Oct; **10** (5): 704-19 http://doi:10.1016/j.sapharm.2-13.11.002

For peer review only

Table 1: Percentage prevalence for burn injury in three rural municipalities in Nepal, 2017- 2018 (n= 1279 Households)

**Percentage responses (rounded to 1 decimal point)**	Indrasarowar, Makwanpur (n=415)	Laxminiya, Dhanusha (n=630)	Ama Chhodingmo, Rasuwa (n=234)	**Total %**	**95 % CI**
at least one burn injury	1.2	3.5	3.0	2.7	1.8,3.7
**who was burned**					
children	0.5	1.4	1.3	1.1	0.6, 1.8
Total adults	0.7	2.1	1.7	1.6	1.0,2.4
women	0.5	1.6	0.9	1.1	1.1, 2.6
men	0.2	0.5	0.9	0.5	0.5, 1.6
**cause of burn**					
flames	0.0	1.7	0.4	0.9	0.5,1.6
scalding	1.0	1.6	1.7	1.4	0.8,2.2
other mechanisms	0.2	0.2	0.9	0.3	0.1,0.8
**where the burn injury occurred**					
at home	1.2	3.5	2.1	2.5	1.7,3.5
away from the home	0.0	0.0	0.9	0.2	0.0,0.7
**location of injury**					
head and neck	0.0	0.5	0.0	0.2	0.0,0.7
limbs	1.0	3.0	2.1	2.2	1.5,3.1
trunk	0.2	0.0	0.9	0.2	0.0,0.7
**whether the injury occurred during cooking**					
while cooking	1.2	3.5	2.1	2.5	1.7,3.5
other activities	0.0	0.0	0.9	0.2	0.0

For peer review only

For peer review only - http://bmjopen.bmj.com/site/about/guidelines.xhtml

Table 2: Burn prevention, first aid and health seeking behaviour in Nepal, 2017- 2018

**Percentage responses (rounded to 1 decimal point)**	Indrasarowar, Makwanpur(n=415)	Laxminiya, Dhanusha(n=630)	Ama Chhodingmo, Rasuwa(n=234)	**Total %**	**95 % CI**
**burn prevention**					
received education on prevention in past 12 months	13.5	10.8	6.8	10.9	9.3,12.8
believe most burns are preventable	52.3	73.8	47.4	62.0	59.3,64.7
believe most people with burns die as a result of their injury	68.0	82.2	70.1	75.4	72.9,77.7
believe that burns can cause bad scars	85.1	96.5	85.5	90.8	89.1,92.3
**first aid**					
received education on first aid in past 12 months	15.7	10.5	5.6	11.3	9.6,13.1
use water	18.3	38.6	13.2	27.4	24.9,29.9
tomato	39.3	5.9	14.1	18.2	16.1,20.4
potato paste	0.0	25.4	0.0	12.5	10.7,14.4
aloe vera	20.0	2.5	1.7	8.1	6.6, 9.7
honey	0.0	13.3	0.0	6.6	5.3,8.1
lotion/tube medicine	5.1	4.6	9.8	5.7	4.5,7.1
other (in order of frequency mango leaf paste, cow dung, mud, herbs, various oils and plants)	5.5	5.9	32.9	10.7	9.1,12.5
would not know what to do	11.8	3.8	28.2	10.9	9.2,12.7
**Clothing catching on fire**					
stop, drop and roll	1.7	5.9	0.0	3.4	2.5,4.6
smother flames with a cloth	13.0	16.7	3.4	13.1	11.3,15
jump in water	7.7	11.6	1.3	8.4	7,10.1
take off the clothing	28.4	53.8	25.6	40.4	37.7,43.2
pour water	34.7	8.7	31.6	21.3	19.1,23.7
would not know what to do	13.0	1.7	36.3	11.7	10, 13.6
**Health seeking behaviour (large or major burn)**					

For peer review only - http://bmjopen.bmj.com/site/about/guidelines.xhtml

For peer review only

Percentage responses (rounded to 1 decimal point)	Indrasarowar, Makwanpur(n=415)	Laxminiya, Dhanusha(n=630)	Ama Chhodingmo, Rasuwa(n=234)	Total %	95 % CI
hospital immediately after incident	67.7	83.8	55.6	73.4	70.9,75.8
primary health care facility* immediately after incident	29.2	15.5	40.6	24.6	22.2,27.0
**Health seeking behaviour (minor burn)**					
manage at home	33.7	37.6	34.6	35.8	33.2, 38.5
primary health care facility*	58.3	58.6	53.0	57.5	54.7,60.2
hospital	1.7	2.4	5.6	2.7	(1.9, 3.8)

*Mainly health post but some pharmacy

Figure 1: Patient journeys, burn injuries in the previous 12 months

Legend: Each participant has been given a unique identifier, colour coded to show the type of injury and the rural municipality where the participant lived. The average, median and mode are given for 1) time of presentation at a health care facility since the injury took place, and 2) the length of stay at the location of treatment.

- to be inserted –

Figure 2: Economic costs and overall impact of burn injuries in the previous 12 months

Legend: Each participant has the same unique identifier as in Figure 1

- to be inserted –

For peer review only

For peer review only - http://bmjopen.bmj.com/site/about/guidelines.xhtml

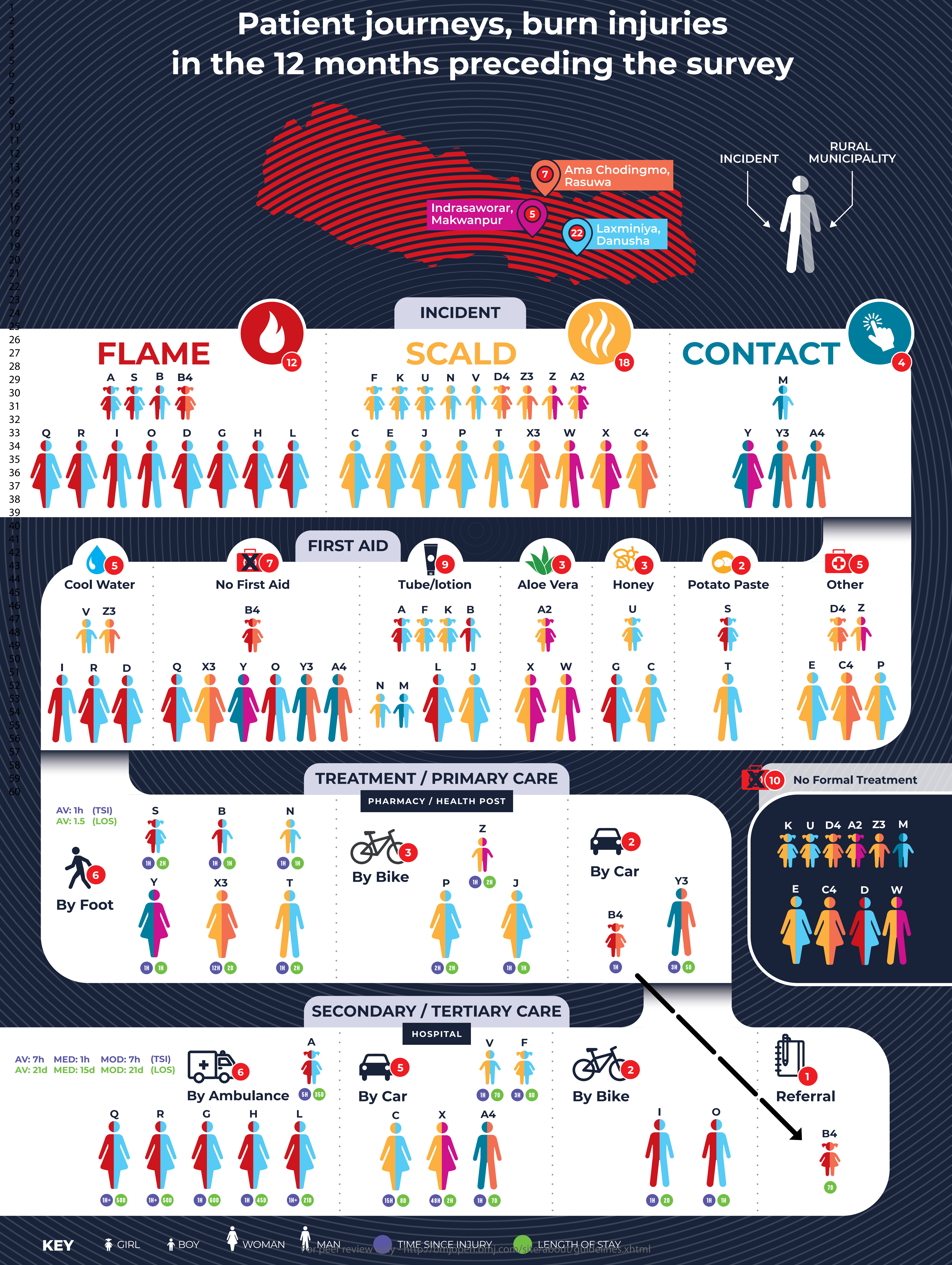

Patient journeys, burn injuries in the 12 months preceding the survey
Ama Chodingmo, Rasuwa
Indrasaworar, Makwanpur
Laxminiya, Danusha
7
5
22
INCIDENT
RURAL MUNICIPALITY
INCIDENT
FLAME
12
SCALD
18
CONTACT
4
FIRST AID
Cool Water 5
No First Aid 7
Tube/lotion 9
Aloe Vera 3
Honey 3
Potato Paste 2
Other 5
TREATMENT / PRIMARY CARE
PHARMACY / HEALTH POST
AV: 1h (TSI)
AV: 1.5 (LOS)
By Foot 6
By Bike 3
By Car 2
No Formal Treatment 10
SECONDARY / TERTIARY CARE
HOSPITAL
AV: 7h MED: 1h MOD: 7h (TSI)
AV: 21d MED: 15d MOD: 21d (LOS)
By Ambulance 6
By Car 5
By Bike 2
Referral 1
KEY
GIRL
BOY
WOMAN
MAN
TIME SINCE INJURY
LENGTH OF STAY

Economic costs and overall impact of burns injuries in 12 months proceeding the survey

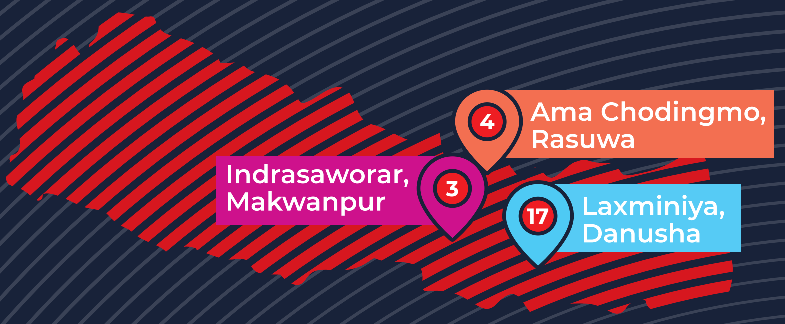

12 FLAME
9 SCALD
3 CONTACT

INCIDENT
RURAL MUNCIPILITY

ECONOMIC COST & IMPACT OF BURN INJURY	IMPACTED INDIVIDUALS	N
Paid more than average cost (NR 4,300/£30) for **primary** care	P, Y	2/10
Paid less than average cost (NR 4,300/£30) for **primary** care	B, J, N, S, T, Q, Z, X3	8/10
Paid more than average daily cost (NR 11,550/£80) for **secondary/tertiary** care	F, Q, R, A4	4/14
Paid less than average daily cost (NR 11,550/£80) for **secondary/tertiary** care	A, C, G, H, I, L, O, V, X, B4	10/14
Borrowed money or sold assets to pay for treatment	A, C, F, G, H, J, L, Q, R, S, T, V, B4	13/24
Reported a major loss of income or educational opportunity	A, C, G, H, L, Q, R, X3, B4	9/24
Reported a minor or no loss of income or educational opportunity	B, F, I, L, O, V, X, N, P, S, T, Y, Z, A4, Y3	15/24
Suffered disability that resulted in full dependence to perform one or more daily tasks (excludes very young children)	A, C, G, L, R, Y3	5/24
Suffered difficulties in relating with others (family and friends) after incident and treatment	C, G	2/24

KEY GIRL BOY WOMAN MAN

For peer review only - http://bmjopen.bmj.com/site/about/guidelines.xhtml

N = 24, WHO RECIEVED FORMAL MEDICAL TREATMENT

STROBE Statement—checklist of items that should be included in reports of observational studies

	Item No	Recommendation
**Title and abstract** **Page 1**	1	(*a*) Indicate the study's design with a commonly used term in the title or the abstract
		(*b*) Provide in the abstract an informative and balanced summary of what was done and what was found
**Introduction**		
Background/rationale Page 3	2	Explain the scientific background and rationale for the investigation being reported
Objectives Page 4	3	State specific objectives, including any prespecified hypotheses
**Methods**		
Study design Page 4	4	Present key elements of study design early in the paper
Setting Page 4; dates page 6	5	Describe the setting, locations, and relevant dates, including periods of recruitment, exposure, follow-up, and data collection
Participants Page 4-5	6	(*a*) *Cohort study*—Give the eligibility criteria, and the sources and methods of selection of participants. Describe methods of follow-up<br>*Case-control study*—Give the eligibility criteria, and the sources and methods of case ascertainment and control selection. Give the rationale for the choice of cases and controls<br>*Cross-sectional study*—Give the eligibility criteria, and the sources and methods of selection of participants
		(*b*) *Cohort study*—For matched studies, give matching criteria and number of exposed and unexposed<br>*Case-control study*—For matched studies, give matching criteria and the number of controls per case
Variables N/A	7	Clearly define all outcomes, exposures, predictors, potential confounders, and effect modifiers. Give diagnostic criteria, if applicable
Data sources/ measurement N/A	8*	For each variable of interest, give sources of data and details of methods of assessment (measurement). Describe comparability of assessment methods if there is more than one group
Bias N/A	9	Describe any efforts to address potential sources of bias
Study size Page 4	10	Explain how the study size was arrived at
Quantitative variables Page 6-7	11	Explain how quantitative variables were handled in the analyses. If applicable, describe which groupings were chosen and why
Statistical methods Page 6	12	(*a*) Describe all statistical methods, including those used to control for confounding
		(*b*) Describe any methods used to examine subgroups and interactions
		(*c*) Explain how missing data were addressed
		(*d*) *Cohort study*—If applicable, explain how loss to follow-up was addressed<br>*Case-control study*—If applicable, explain how matching of cases and controls was addressed<br>*Cross-sectional study*—If applicable, describe analytical methods taking account of sampling strategy
		(*e*) Describe any sensitivity analyses

For peer review only

For peer review only

**Results**		
Participants<br>Page 7	13*	(a) Report numbers of individuals at each stage of study—eg numbers potentially eligible, examined for eligibility, confirmed eligible, included in the study, completing follow-up, and analysed
		(b) Give reasons for non-participation at each stage
		(c) Consider use of a flow diagram
Descriptive data<br>Page 7	14*	(a) Give characteristics of study participants (eg demographic, clinical, social) and information on exposures and potential confounders
		(b) Indicate number of participants with missing data for each variable of interest
		(c) *Cohort study*—Summarise follow-up time (eg, average and total amount)
Outcome data<br>N/A	15*	*Cohort study*—Report numbers of outcome events or summary measures over time
		Case-control study—Report numbers in each exposure category, or summary measures of exposure
		Cross-sectional study—Report numbers of outcome events or summary measures
Main results<br>N/A	16	(*a*) Give unadjusted estimates and, if applicable, confounder-adjusted estimates and their precision (eg, 95% confidence interval). Make clear which confounders were adjusted for and why they were included
		(*b*) Report category boundaries when continuous variables were categorized
		(*c*) If relevant, consider translating estimates of relative risk into absolute risk for a meaningful time period
Other analyses<br>N/A	17	Report other analyses done—eg analyses of subgroups and interactions, and sensitivity analyses
**Discussion**		
Key results<br>Page 9	18	Summarise key results with reference to study objectives
Limitations<br>Page 10	19	Discuss limitations of the study, taking into account sources of potential bias or imprecision. Discuss both direction and magnitude of any potential bias
Interpretation<br>Page 10	20	Give a cautious overall interpretation of results considering objectives, limitations, multiplicity of analyses, results from similar studies, and other relevant evidence
Generalisability<br>Page 10	21	Discuss the generalisability (external validity) of the study results
**Other information**		
Funding<br>Page 11	22	Give the source of funding and the role of the funders for the present study and, if applicable, for the original study on which the present article is based

*Give information separately for cases and controls in case-control studies and, if applicable, for exposed and unexposed groups in cohort and cross-sectional studies.

Note: An Explanation and Elaboration article discusses each checklist item and gives methodological background and published examples of transparent reporting. The STROBE checklist is best used in conjunction with this article (freely available on the Web sites of PLoS Medicine at http://www.plosmedicine.org/, Annals of Internal Medicine at http://www.annals.org/, and Epidemiology at http://www.epidem.com/). Information on the STROBE Initiative is available at www.strobe-statement.org.

BMJ Open

Burns in Nepal: a participatory, community survey of burn cases and knowledge, attitudes and practices to burn care and prevention in 3 rural municipalities

Journal:	*BMJ Open*
Manuscript ID	bmjopen-2019-033071.R3
Article Type:	Original research
Date Submitted by the Author:	03-Feb-2020
Complete List of Authors:	Phuyal, Kamal; SAGUN: a search for harmony<br>Ogada, Edna; Swansea University, Centre for Global Burn Injury Policy and Research, College of Human and Health Sciences<br>Bendell, Richard; Interburns<br>Price, Patricia; Swansea University; Interburns<br>Potokar, Tom; Swansea University, Centre for Global Burn Injury, Policy and Research, College of Human and Health Sciences; InterBurns
**Primary Subject Heading**:	Public health
Secondary Subject Heading:	Epidemiology
Keywords:	Burns, Prevention, Community

SCHOLARONE™
Manuscripts

I, the Submitting Author has the right to grant and does grant on behalf of all authors of the Work (as defined in the below author licence), an exclusive licence and/or a non-exclusive licence for contributions from authors who are: i) UK Crown employees; ii) where BMJ has agreed a CC-BY licence shall apply, and/or iii) in accordance with the terms applicable for US Federal Government officers or employees acting as part of their official duties; on a worldwide, perpetual, irrevocable, royalty-free basis to BMJ Publishing Group Ltd ("BMJ") its licensees and where the relevant Journal is co-owned by BMJ to the co-owners of the Journal, to publish the Work in this journal and any other BMJ products and to exploit all rights, as set out in our licence.

The Submitting Author accepts and understands that any supply made under these terms is made by BMJ to the Submitting Author unless you are acting as an employee on behalf of your employer or a postgraduate student of an affiliated institution which is paying any applicable article publishing charge ("APC") for Open Access articles. Where the Submitting Author wishes to make the Work available on an Open Access basis (and intends to pay the relevant APC), the terms of reuse of such Open Access shall be governed by a Creative Commons licence – details of these licences and which Creative Commons licence will apply to this Work are set out in our licence referred to above.

Other than as permitted in any relevant BMJ Author's Self Archiving Policies, I confirm this Work has not been accepted for publication elsewhere, is not being considered for publication elsewhere and does not duplicate material already published. I confirm all authors consent to publication of this Work and authorise the granting of this licence.

For peer review only

Title: *Burns in Nepal: a participatory, community survey of burn cases and knowledge, attitudes and practices to burn care and prevention in 3 rural municipalities*

Authors:

Kamal Phuyal[1], Edna Ogada[2], Richard Bendell[3], Patricia Price [2,3], Tom Potokar [2,3]

1. SAGUN: a search for harmony; Kupandol, Lalitpur, Nepal
2. Centre for Global Burn injury Policy and Research, Swansea University
3. InterBurns, Welsh Centre for Burns and Plastic Surgery, Swansea

Corresponding author:

Professor Patricia Price,

Centre for Global Burn injury Policy and Research,

College of Human and Health Sciences

Room 26 Haldane Building

Swansea University

Singleton Park

Swansea SA2 8PP

Email: p.e.price@swansea.ac.uk

Keywords:

Burns/epidemiology: Burns/prevention: Community participation: Survey: First Aid

For peer review only

Abstract

Objectives: As part of an on-going, long-term project to co-create burn prevention strategies in Nepal, we collected baseline data to share and discuss with the local community, use as a basis for a co-created prevention strategy and then monitor changes over time. This paper reports on the method and outcomes of the baseline survey and demonstrates how the data are presented back to the community.

Design: a community-based survey

Setting: Community based in 3 Rural Municipalities in Nepal.

Participants: 1305 households were approached: the head of 1279 households participated, giving a response rate of 98%. In 90.3% of cases the head of the household was male.

Results

We found that 2.7% (CI: 1.8, 3.7) of 1279 households, from 3 representative municipalities, reported at least one serious burn in the previous 12 months: a serious burn was defined as one requiring medical attention and/or inability to work or do normal activities for 24 hours. Whilst only 4 paediatric and 10 adult cases in the previous 12 months reached hospital care, the impact on the lives of those involved was profound. Only one patient was referred on from primary to secondary/tertiary care; the average length of hospital stay for those presenting directly to secondary/tertiary care was 21 days. A range of first aid behaviours were used, many of which are appropriate for the local context whilst a few may be potentially harmful (e.g., the use of dung).

Conclusion

The participatory approach used in the study ensured a high response rate. We have demonstrated that infographics can link the pathway for each of the cases observed from initial incident to final location of care.

Article Summary:

Strengths

- Detailed and sustained work with the local community to ensure enhanced community participation in the survey
- Tracking individual patient journeys from injury to final destination for burn care
- The use of info-graphics to overcome language and knowledge barriers when presenting the findings back to the community.

Limitations

- Only one person with a burn injury per household was included in the study.

For peer review only

Introduction

The World Health Organisation has repeatedly tried to raise awareness of the fact that the majority of the burden of burn injury, from a global perspective, is borne by low and middle-income countries [1,2]. Other recent publications[3] have confirmed that the greater burden of mortality and morbidity related to burn injury is borne by the world's poor, residing mostly in LMICs. Patients in these countries continue to endure an extremely high level of suffering, with the rate of child deaths from burns being over 7 times higher in lower resource settings than in the western environment[4,5]. Mongolia has a particularly high rate of child burn injury as identified by The Global Burden of Disease study in 2013[5]; detailed community studies, conducted in Ulaanbaatar, have supported this finding and shown that 27% (N=247/900) had a history of burn injury[6]. A recent systematic review[7] found eight epidemiological studies based in Nepal and concluded that burn injuries are the leading cause of morbidity and mortality in the country. The study authors report mortality rates from 4.5% to 23.5% and conclude that the limited data explains the lack of effective intervention programmes in the area.

The Annual Report from the Department of Health and Population for Nepal, covering the fiscal year 2016-17, recorded 55,090 burn or scald injuries nationally[8]. Population statistics for the same time period were 28.9M, giving an incidence rate of 0.19% of patients with an injury sufficiently severe to seek help from an outpatient facility where data were recorded. Data from public hospitals (92% response rate) and private facilities (47.2% response rate) submitted as part of the Annual Report[8] and using the ICD codes T200 – T301 for inpatients, 1710 cases were reported with 1006 related to those below the age of 19, and 586 under the age of 5 (81.7% of these were aged 1-4). In the same time period there were 46 in-hospital deaths reported (31 F, 15 M) – although this does not include deaths from catastrophic events or from those who were sent straight to the mortuary.

Understanding the true morbidity and mortality impacts of burn injury in Nepal is challenging. Gupta et al[9] conducted a household survey using a cluster randomised cross section country wide survey in 15 districts in Nepal and reported a higher incidence of 55 burns from 1350 households (2%, 95% CI 1.5-2.6%). The majority of cases (60.4%) were due to hot liquid and/or hot objects. The study authors conclude that the demographics and aetiology of burns at a population level vary significantly from hospital level data. Tripathee and Basner[7], in their systematic review, stress the importance of epidemiological data as a pre-requisite for planning and implementing prevention programmes at the community level.

For peer review only - http://bmjopen.bmj.com/site/about/guidelines.xhtml

To proactively develop lasting changes in resource poor countries, it is vital that we have a detailed understanding of the current, local context including epidemiology, the availability of educational/prevention programmes and the ability of people in rural/remote areas to access vital first aid treatment in order to plan appropriate interventions. This paper focuses on the first part of a major initiative to address these questions and bring about change using an Implementation Science approach, based in Nepal. Whilst previous studies start to give us an indication of the scale of the problem, they do not give us information about the ways in which people sought help, what they did immediately after the injury, what local knowledge exists to provide first-aid and what prevention programmes are available locally. In order to develop a baseline for future comparison and ensure that we could fully evaluate our interventions, we needed to conduct a more in-depth community based survey so that we could develop and co-create (with community stakeholders) a community prevention and first aid strategy. This paper presents the findings of the baseline phase of the study. The specific objectives, related to selected areas of Nepal, were to: –

i. record the incidence and causes of burn injury in 2017-18,

ii. explore the knowledge, attitudes and practices of the community regarding burn care and prevention.

Methods

The on-going study uses a participatory action research design to develop a prevention and first aid strategy at the rural municipality level (RM) (n= 460 RM in Nepal). This first phase included a community sensitization and preparation plan, followed by baseline data collection. As the prevention strategies need to be developed at the rural municipality level, baseline data was collected in one randomly selected municipality in each of three districts, Ama Chhodingmo Rural Municipality (ARM) in Rasuwa, Indrasarowar Rural Municipality (IRM) in Makawanpur and Laxminiya Rural Municipality (LRM) in Dhanusha. These districts were drawn from three traditionally noted geographic regions of Nepal, Terai (flatland), Hills, and Himalayan regions. This purposive selection was used to ensure a degree of diversity in the communities studied, whilst representing the geo-political areas that any national policy would need to address.

The study population was all the people living in those rural municipalities, regardless of socio-demographic factors. A sample of 1305 households was selected from a population of 47,642, based on the WHO formula[10], adjusted to account for the relative population size in each district. Household selection was completed using a systematic sampling approach drawing from a listing of the total

For peer review only - http://bmjopen.bmj.com/site/about/guidelines.xhtml

number of households in each RM, formulated during the sensitisation preparation phase at each municipality office, when detailed discussions took place with local stakeholders.

Rasuwa district is located north of Kathmandu. It is a district of natural resources, particularly Langtang and Gosaikunda regions, which are well known for their natural beauty and trekking routes, with a high potential for eco-tourism businesses. However, poor roads, poor access to university education and poverty have hindered the development of the district. Ama Chhodingmo is located in the mountain region, bordering China to the north, and contains both natural and cultural diversity. The landscape in this area makes road construction very difficult, and most of the northern areas lack access to vehicle-friendly roads. Rasuwa itself is accessible by bus from Kathmandu via the Pasang Lhamu highway, but the administrative headquarters (Dhunche) are about 120 km from the capital. Over 98% of the population are Hill Ethnic Groups or Tamang; this is one of the indigenous inhabitant groups of Nepal, with their own distinct language, living in scattered hill villages.

Makawanpur is a hilly district, located on the southern border of Kathmandu. Despite bordering Kathmandu valley, many areas of Makwanpur remain inaccessible by road. The main livelihood for the majority of the population is subsistence farming. The district includes within its borders various agro-climatic zones: valleys, plains, and mid-to-high hills. High rates of unemployment have driven a surge of migration - particularly of younger people - to Kathmandu and abroad. Indrasarowar rural municipality, selected for this project, is located in the north-eastern part of the district in which the majority of the population belong to Tamang ethnic group (80%). Just over 9% of the population belong to the Newar ethnic group, who are the historical inhabitants of the Kathmandu Valley, while a further 9.6% of the population are Hill Brahmin/Chhetri. Just over 70% of the population speak Tamang, with 26.5% speaking Nepali.

Dhanusha is one of the southern Terai districts of Janakpur Zone and is an agriculture dependent district. Janakpurdham city is the capital of State 2 where this district is located and one of the commercial centres in the Terai region. It is also the administrative hub of the state. Laxminiya rural municipality is located around 5 km from the major city of Janakpur; the settlement straddles a highway, allowing for some road access. The main livelihood is subsistence farming. The majority of people in this rural municipality belong to Madhesi middle caste (i.e. Yadav, Sudhi, Lohar, Sonar; this is a new term recently accepted into official vocabulary to describe this separate group in the Nepali social classification system) and Maithili is their main language. This is the most densely populated area included in the study.

For peer review only - http://bmjopen.bmj.com/site/about/guidelines.xhtml

Initial discussions with the key stakeholders included 15 Key Informant Interviews (KIIs) and 19 Focus Group Discussions (FGDs) with Female Community Health Volunteers (FCHV), teachers, social workers and health care staff. This important first stage of the study, which took place over a six-month period in the first half of 2018, allowed the researchers to develop their understanding of the general context of the burn injuries. Participant observation sessions (n=13) that focused on food storage and cooking practices, together with social maps and seasonal calendars, were used to augment the narrative on burn injuries, their prevention and management in each of the rural municipalities. Based on this initial work, items for the survey were prepared both in Nepali and English and reviewed by 5 local experts (including Sagun board members and university staff) to enhance their validity. The data collection tool asked households to report on any burn injury that required medical attention (which could include a medical post, a health worker, a Female Community Health Volunteer, or a pharmacy) in the previous 12 months.

Survey data was collected in July and August 2018 using a structured questionnaire to collect data on social demographic and household descriptors, incidences of burn injury meeting the case definition, first aid received and health seeking behaviour after the burn injury. The questionnaire was pre-tested in Mangaltar of Kavre district, where it was piloted on 15 households; only minor changes were required at the end of this phase. The final version of the survey tool was developed into an electronic format (CSPro software) in order to assist with the logistics of collecting data in remote areas, where smart phones are used routinely despite the geographic remoteness.

All enumerators, from a pool of experienced participatory research data collectors regularly used by Sagun, were trained in data collection using an electronic version of the form on smart phones to ensure consistency of use and reduce human error. The training included a day on theory and a day on practical issues, followed up by 3 supervised community-based training opportunities resulting in 3-5 full days of supervised training. All enumerators were instructed to call at the selected households up to 3 times until someone was present to complete the questionnaire: after 3 attempts no further attempt was made to contact the household members.

Three introductory visits were organised in each RM to discuss the details of the research proposal with representatives of the local government, health care staff and other relevant stakeholders at the RM level. A letter of approval was provided by each of the relevant local governments. The research proposal was reviewed and approved by Nepal Health Research Council (NHRC-Reg. no.348/2018),

For peer review only - http://bmjopen.bmj.com/site/about/guidelines.xhtml

with written consent taken from each household. Survey data was recorded and analysed using IBM SPSS version 23. The unit of analysis was taken to be 'the household'.

The majority of the analysis was descriptive in nature and involved summarising findings by region, gender, type of burn, initial first aid and final destination of health service need. The data are presented for the whole cohort, as well as by region. The regional data are important to ensure the range of local experiences are reflected appropriately: the data for the whole cohort are important in order to provide policy makers with the relevant information for national strategies. Our experiences in the area have highlighted that the local communities feel that they are frequently being asked to participate in surveys – yet feel that nothing changes. So, although it is important to fully understand the baseline situation so that we can evaluate whether our future interventions have been effective, we have also sought ways of presenting our findings to the local community that are easy to understand across languages. The focus has been to highlight the ways in which people access burn care by understanding their pathway from injury to treatment as a first step in co-creating prevention strategies that are locally relevant and implementable.

Patient and Public Involvement

The public and former patients were involved in the design of the study through interviews with key personnel, by allowing the researchers to observe their everyday practices (for example, routine cooking and food storage practices) and through community focus groups (n=19). The key stakeholders involved included political leaders, health post workers, household members (including burn survivors), women's groups, and schoolteachers. These same groups were involved in the direct dissemination of the community findings using info-grams and case-based narratives, and will help to develop locally relevant and locally owned awareness campaigns, prevention strategies and educational materials to support appropriate first-aid practices for the next phase of the study.

Results

1305 households were included in the original sampling frame; data were recorded from 1279 (98% of the initial sample). In the 12 months preceding the survey, 2.7% of the households reported at least one serious burn injury (see Table 1). Of the three RM's, Laxminiya reported 4 of every 100 households experienced a burn injury, Indrasarowar reported 1:100, while Ama Chhodingmo reported 3:100. More women were affected, scalds being the most frequent cause of injury and the injury was most likely to happen at home; more injuries were reported in adults than children over the 12-month time frame.

For peer review only - http://bmjopen.bmj.com/site/about/guidelines.xhtml

Table 2 presents descriptive data on knowledge and practices on burn prevention, first aid and health seeking behaviour in all 1279 households. Exposure to prevention education was limited, with only one in ten households self-reported exposure to education or information about first aid treatment for burns. Two-thirds of the households indicated that they believe that most burns were preventable, with a significantly greater proportion (74%) in Laxminiya; interestingly, this is where the rates for burn injury in the last 12 months were the highest.

The most frequent response to first aid response was the use of water when a family member was scalded (27.4%), with the application of lotion as the lowest response (5.7%). However, Table 2 also outlines regional variations with Laximiniya reporting the highest use of water (38.6%) whereas the highest response in Indrasarowar was to apply tomato, and the highest response in Ama Chhodingmo was to use a range of other options (including mango leaf paste, cow dung, mud, and herbs). Households in Ama Chhodingmo also reported that 28.2% say they would not know what to do in such circumstances.

Forty percent would take off clothing if they caught fire (40.4%); 3 in 10 households reported they would either pour water onto the victim or jump into water. Only 3 in every 100 households reported that they would stop, drop and roll, while about 1 in 10 reported they would smother the flames with a cloth. One out of every 10 households indicated they would not know what to do. These overall figures again hide substantial differences in regional responses (Table 2).

The majority of Households responded that they would take a family member to hospital following a major burn (73.4%) or to a primary healthcare facility for a minor burn (57.5%). There were regional differences, with only a little over half of households in Ama Chhodingmo reporting they would take a family member to hospital immediately after a large or major burn (55.6%). One third of all households reported that they would manage minor burn incidents at home immediately after the incident, which was consistently reported across the three study sites.

The data relating the experiences of those injured in the previous 12 months have been summarised in an info-gram (Figure 1) which illustrates patient journeys from initial injury to treatment; each identified case from the data base has been given an icon to reflect whether they are male or female, and an adult or a child. The left hand side of the icon reflects the type of burn injury, with the right hand side reflecting their rural municipality. For example, Case A is a female child from Laximinya, who

For peer review only - http://bmjopen.bmj.com/site/about/guidelines.xhtml

experienced a flame burn, was treated initially with an alternative form of first aid (tube/lotion) and was transferred by ambulance 5 hours after injury and then spent 35 days in hospital. Ten reported cases did not seek any formal treatment beyond first aid; eleven cases reported to a primary care facility, one of which (B4) was referred onto secondary/tertiary care where she stayed for 7 days; the remaining 13 cases presented directly to secondary/tertiary care where the average length of hospital stay was 21 days.

Figure 1 shows that only 5 out of 34 burns received appropriate first aid in the form of cooling the wound prior to applying alternative methods for first aid. One in 4.4 burn victims applied some form of tube based lotion or medicine as a first aid measure indicating the availability of some form of 'over the counter' burn remedy. Minor burns that can be managed at health posts or pharmacies were filtered appropriately by self-referral, while major or serious burns presented to secondary or tertiary hospitals. The data indicate that of the most serious burn cases requiring admission are disproportionately experienced by women/girls; in all cases they are linked to cooking activities. Figure 2, which summarises the economic and social costs for the 24 individuals who received formal treatment, shows that more than half reported having to borrow money or sell assets to cover the costs of their treatment. One in four suffered a disability that resulted in full dependence to perform one or more daily tasks.

Discussion

Our data on self-report of serious burn injury, requiring secondary or tertiary care are substantially higher than we anticipated, compared to the Annual Report[8], and reflect a substantial number of households that have experienced a serious burn, many of whom needed hospital care and will likely need on-going management for their pain, disabilities and limitations in function, and ability to be self-sufficient. However, responses from Ama Chhodingmo suggested that only a half of households would take serious cases directly to hospital, which may be a reflection of lack of access in terms of resources - financial and otherwise, or physical distance to secondary or tertiary level services.

Together with the background information collected in the initial phases of the study, the survey provides great insight into the risk factors and prevailing knowledge of burn injury at the rural municipality level, as well as the personal financial costs borne as a consequence of burn injuries. Presenting this information in a simple visual format to local stakeholder groups will enable informed discussion and support the co-creation approach that will empower local communities to implement prevention activities.

For peer review only - http://bmjopen.bmj.com/site/about/guidelines.xhtml

Our ability to plot the experiences of individuals from initial injury through to length of hospital stay, will allow local health teams to work in tandem with local communities to fully explain the importance of initial first aid and the consequences of delayed (or inappropriate) treatment. The use of info-grams, to support patient understanding of health messages[11], has enabled us to summarise the data in an easily digestible format and will allows us to work with a wide range of stakeholders. Although some translation will be required, the emphasis on imagery should support us to disseminate messages to health care professionals across the country. Initial work on scoping out a prevention strategy has already highlighted the importance of this local knowledge when interpreting the data, and of infograms as a focal point for meaningful discussions when co-creating a context-relevant prevention strategy.

Our data suggest that the key areas for prevention would be cooking within the home setting, particularly for women and children. These data are supported by previous studies included in a systematic review[7]. These authors reported that five out of the eight studies reviewed found more burns in females than males, and the most common location for a burn to occur was inside the house, mostly in the kitchen. One study included in the review reported that 86% of childhood burns occurred in the home. Local knowledge will be extremely important in identifying how this information needs to be built into a program. First aid messages need to stress the importance of cool clean water as an initial treatment, when it is available (particularly for scald and flame burns); however, programs will need to reflect on what is locally available and differentiate between acceptable, alternative cooling options and those that must be avoided (e.g., mud). When clothing catches fire, household members need to be aware of the stop, drop and roll method and prioritise that no additional body parts (or other individuals or items) catch fire in the process.

This was a robustly designed study with a strong emphasis on using local knowledge to inform the content of the data collection tool, and substantial emphasis was placed on spending time with the local communities to ensure their full involvement. However, it is important not to over interpret the findings of the survey at either district or national level; any method that relies on recall is subject to memory bias that can lead to inaccuracies in how the original incident was reported in the survey. In this study, the proportional distribution of households recruited roughly reflected the relative population sizes of the RM's in each district. Only one member of each household, even in households with multiple burn injuries, was interviewed about the incident; the individual selected was the one with the most serious injury meeting the case definition. Given that many households in Nepal are

For peer review only

multi-generational and large in size, the person interviewed may not have been aware of all burn incidents within the household so the possibility exists that on some occasions there may have been a more severe incident within the household that was not captured in this data set. This approach may have resulted in a potential underestimation of burn injuries and any new studies should consider interviewing all those with a serious burn injury.

The team involved in the initial stages of the project are already working, in collaboration with the research team, to co-create appropriate prevention strategies including a wide range of locally relevant activities, where the local community propose ideas that would make a real difference for their own communities. This is an important initial step in developing locally relevant and locally owned prevention strategies to help reduce the incidence of burns, whilst ensuring that those with an injury receive the most appropriate first aid and seek appropriate care as quickly as possible.

Acknowledgements

We would especially like to acknowledge the support of Mr Bindeshwor Yadav, Chairperson, Laxminiya Rural Municipality, Dhanusha; Mr Buchung Tamang, Chairperson, Ama Chhodingmo Rural Municipality, Rasuwa; and Mr Jivan Lama, Chairperson, Indrasarowar Rural Municipality, Makawanpur.

With thanks to Arjun Bhattarai for his support in developing the online tool for data capture and support with the sampling frame.

The project team would like to express their heartfelt thanks to everyone who took the time to participate in the survey and to the local SAGUN team members who are so enthusiastic to bring about change and improvements in their local communities in Nepal.

Contribution of authors

Kamal Phuyal: Local lead for SAGUN in Nepal and the local research contact for the survey

Edna Ogada: Research assistant with responsibility for assisting with the ethical approval process, organising and analysing the data

Richard Bendell: Program manager for InterBurns, supported the logistics for the project and the design of the data collection tool

For peer review only - http://bmjopen.bmj.com/site/about/guidelines.xhtml

Patricia Price: Academic lead and responsible for writing the paper

Tom Potokar: Grant holder and Chief Investigator.

All authors have reviewed the paper prior to submission

Competing Interests

There are no competing interests for any of the authors.

Funding

This project is funded by the National Institute for Health Research (NIHR), NIHR Global Health Research Group on Burn Trauma, Grant Reference 16/137/110. The views expressed are those of the authors and not necessarily those of the NIHR of the Department of Health and Social Care.

Data Sharing

Data will be available from NIHR Open Data supplied by NIHR Centre for Business Intelligence, Crown Copyright and database licensed under the Open Government Licence (http://www.nationalarchives.gov.uk/doc/open-government-licence/version/3/): Global Health Research (in development). Data will also be archived at Swansea University.

References

1. Peck M, Molnar J & Swart D. A global plan for burn prevention and care. *Bulletin of the World Health Organisation* 2009;**87**: 802-803 doi: 10.2471/BLT.08.059733
2. WHO Factsheet on Burns: March 2018 (accessed May 7 2019) available at http://www.who.int/news-room/fact-sheets/detail/burns
3. Stokes, MAR & Johnson WD. Burns in the Third World: an unmet need. *Annals of Burns Fire Disasters*. 2017 Dec 31; **30**(4): 243–246.
4. Mathers CD, Bernard C, Moesgaard Iburg K, *et al*. 2003. Global Burden of Disease in 2002: data sources, methods and results (Version 2). *Global Programme on Evidence for Health Policy Discussion* Paper No.**54**. World Health Organisation; Geneva http://www.who.int/healthinfo/paper54.pdf
5. Sengoelge M, El-Khatib Z & Laflamme L. The global burden of child burn injuries in the light of country level economic development. *Preventive Medicine Reports* 2017; **6**: 115-120 http://dx.doi.org/10.1016/j.p.medr.2017.02.024

For peer review only - http://bmjopen.bmj.com/site/about/guidelines.xhtml

6. Public Health Institute. *Survey on risk factors for burn injuries in 0-5 year old children and related knowledge, attitudes and practices of their caregivers.* Ulaanbaatar: Ministry of Health of Mongolia 2009

7. Tripathee S & Basnet SJ Epidemiology of burn injuries in Nepal: a systematic review. *Burns and Trauma* 2017 **5**:10 doi 10.1186/s41038-017-0075-y

8. Ministry of Health and Population. *Annual Report,* 2016. Department of Health Services, Kathmandu, Nepal

9. Gupta S, Mahmood U, Gurung S, et al. Burns in Nepal: A population based national assessment. *Burns* 2015 **41**(5): 1126-1132 doi:10.1016/j.burns.2014.11.012

10. Sethi D, Habibula S, McGee K, *et al*. (Eds) *Guidelines for Conducting Community Surveys on Injuries and Violence.* 2004 World Health Organisation, Geneva ISBN: 92 4 154 648 4

11. Barros IM, Alcantara TS, Mesquita M *et al* The use of pictograms in health care: a literature review. *Res Social Admi Pharm* 2014, Sept-Oct; **10** (5): 704-19 http://doi:10.1016/j.sapharm.2-13.11.002

For peer review only

Table 1: Percentage prevalence for burn injury in three rural municipalities in Nepal, 2017- 2018 (n= 1279 Households)

**Percentage responses***	Indrasarowar, Makwanpur (n=415)	Laxminiya, Dhanusha (n=630)	Ama Chhodingmo, Rasuwa (n=234)	**Total %**	**95 % CI**
at least one burn injury	1.2	3.5	3.0	2.7	1.8,3.7
**who was burned**					
children	0.5	1.4	1.3	1.1	0.6, 1.8
Total adults	0.7	2.1	1.7	1.6	1.0,2.4
women	0.5	1.6	0.9	1.1	1.1, 2.6
men	0.2	0.5	0.9	0.5	0.5, 1.6
**cause of burn**					
flames	0.0	1.7	0.4	0.9	0.5,1.6
scalding	1.0	1.6	1.7	1.4	0.8,2.2
other mechanisms	0.2	0.2	0.9	0.3	0.1,0.8
**where the burn injury occurred**					
at home	1.2	3.5	2.1	2.5	1.7,3.5
away from the home	0.0	0.0	0.9	0.2	0.0,0.7
**location of injury**					
head and neck	0.0	0.5	0.0	0.2	0.0,0.7
limbs	1.0	3.0	2.1	2.2	1.5,3.1
trunk	0.2	0.0	0.9	0.2	0.0,0.7
**whether the injury occurred during cooking**					
while cooking	1.2	3.5	2.1	2.5	1.7,3.5
other activities	0.0	0.0	0.9	0.2	0.0

* Figures have been rounded to one decimal place

For peer review only

Table 2: Burn prevention, first aid and health seeking behaviour in Nepal, 2017- 2018

**Percentage responses***	Indrasarowar, Makwanpur (n=415)	Laxminiya, Dhanusha (n=630)	Ama Chhodingmo, Rasuwa (n=234)	**Total %**	**95 % CI**
**burn prevention**					
received education on prevention in past 12 months	13.5	10.8	6.8	10.9	9.3,12.8
believe most burns are preventable	52.3	73.8	47.4	62.0	59.3,64.7
believe most people with burns die as a result of their injury	68.0	82.2	70.1	75.4	72.9,77.7
believe that burns can cause bad scars	85.1	96.5	85.5	90.8	89.1,92.3
**received first aid education**					
received education on first aid in past 12 months	15.7	10.5	5.6	11.3	9.6,13.1
**first aid**					
use water	18.3	38.6	13.2	27.4	24.9,29.9
tomato	39.3	5.9	14.1	18.2	16.1,20.4
potato paste	0.0	25.4	0.0	12.5	10.7,14.4
aloe vera	20.0	2.5	1.7	8.1	6.6, 9.7
honey	0.0	13.3	0.0	6.6	5.3,8.1
lotion/tube medicine	5.1	4.6	9.8	5.7	4.5,7.1
other (in order of frequency mango leaf paste, cow dung, mud, herbs, various oils and plants)	5.5	5.9	32.9	10.7	9.1,12.5
reported that they would not know what to do	11.8	3.8	28.2	10.9	9.2,12.7
**Clothing catching on fire**					
stop, drop and roll	1.7	5.9	0.0	3.4	2.5,4.6
smother flames with a cloth	13.0	16.7	3.4	13.1	11.3,15
jump in water	7.7	11.6	1.3	8.4	7,10.1
take off the clothing	28.4	53.8	25.6	40.4	37.7,43.2
pour water	34.7	8.7	31.6	21.3	19.1,23.7
reported that they would not know what to do	13.0	1.7	36.3	11.7	10, 13.6

For peer review only - http://bmjopen.bmj.com/site/about/guidelines.xhtml

Percentage responses*	Indrasarowar, Makwanpur (n=415)	Laxminiya, Dhanusha (n=630)	Ama Chhodingmo, Rasuwa (n=234)	Total %	95 % CI
**Health seeking behaviour (large or major burn)**					
hospital immediately after incident	67.7	83.8	55.6	73.4	70.9,75.8
primary health care facility** immediately after incident	29.2	15.5	40.6	24.6	22.2,27.0
**Health seeking behaviour (minor burn)**					
manage at home	33.7	37.6	34.6	35.8	33.2, 38.5
primary health care facility*	58.3	58.6	53.0	57.5	54.7,60.2
hospital	1.7	2.4	5.6	2.7	(1.9, 3.8)

*Figures have been rounded to one decimal place

**Mainly health post but some pharmacy

Figure 1: Patient journeys, burn injuries in the previous 12 months

Legend: Each participant has been given a unique identifier, colour coded to show the type of injury and the rural municipality where the participant lived. The average, median and mode are given for 1) time of presentation at a health care facility since the injury took place, and 2) the length of stay at the location of treatment.

- to be inserted –

Figure 2: Economic costs and overall impact of burn injuries in the previous 12 months

Legend: Each participant has the same unique identifier as in Figure 1

- to be inserted –

For peer review only

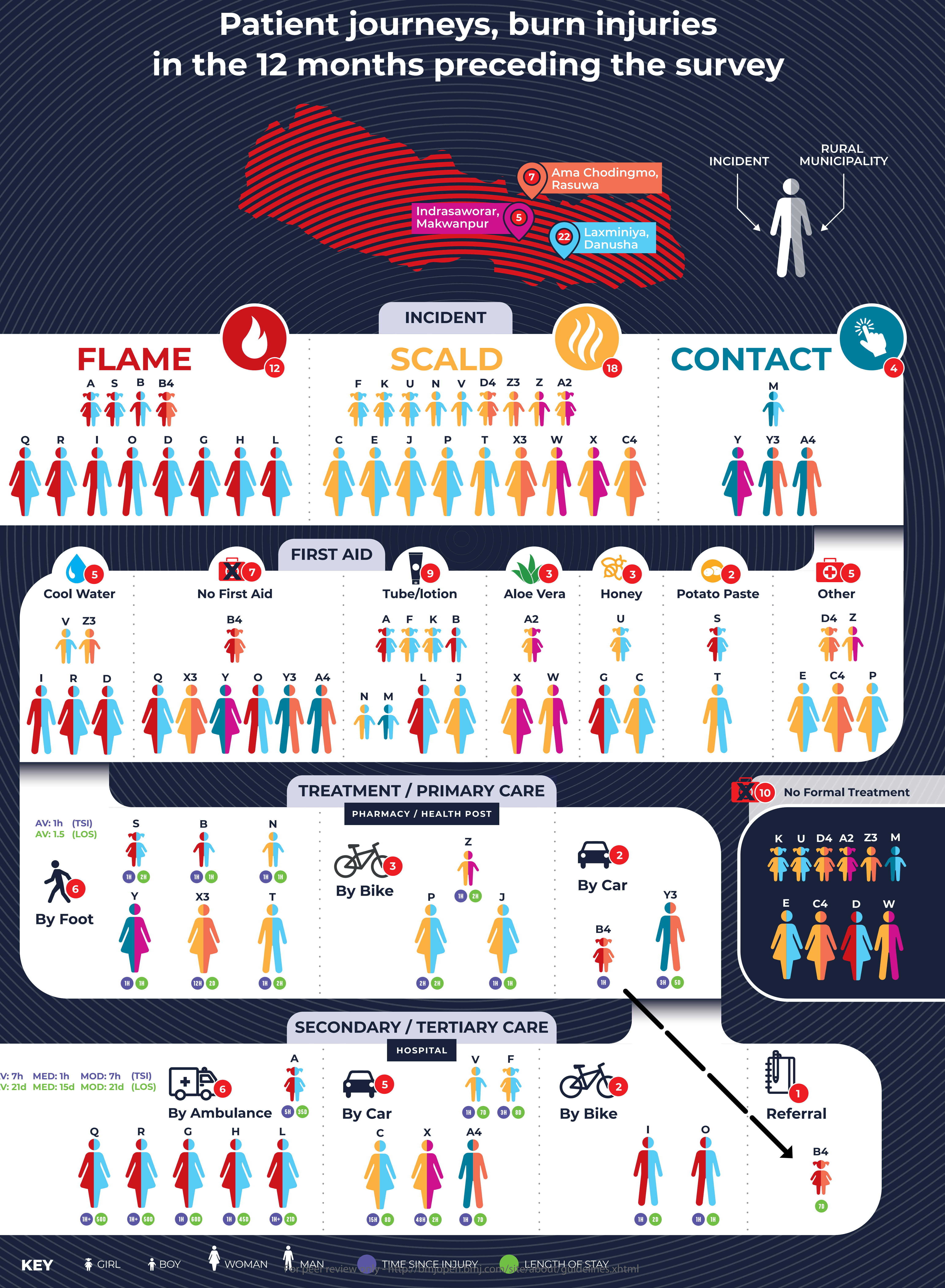
Patient journeys, burn injuries
in the 12 months preceding the survey
Ama Chodingmo, Rasuwa
Indrasaworar, Makwanpur
Laxminiya, Danusha
INCIDENT
RURAL MUNICIPALITY
INCIDENT
FLAME
SCALD
CONTACT
FIRST AID
Cool Water
No First Aid
Tube/lotion
Aloe Vera
Honey
Potato Paste
Other
TREATMENT / PRIMARY CARE
PHARMACY / HEALTH POST
No Formal Treatment
AV: 1h (TSI)
AV: 1.5 (LOS)
By Foot
By Bike
By Car
SECONDARY / TERTIARY CARE
HOSPITAL
AV: 7h MED: 1h MOD: 7h (TSI)
AV: 21d MED: 15d MOD: 21d (LOS)
By Ambulance
By Car
By Bike
Referral
KEY
GIRL
BOY
WOMAN
MAN
TIME SINCE INJURY
LENGTH OF STAY

Economic costs and overall impact of burns injuries in 12 months proceeding the survey

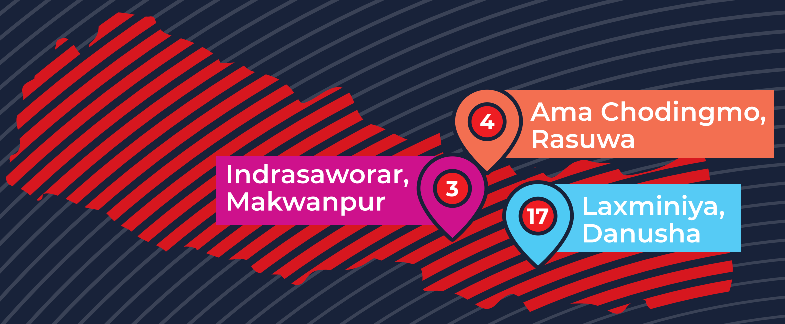

12 FLAME
9 SCALD
3 CONTACT

INCIDENT — RURAL MUNCIPILITY

ECONOMIC COST & IMPACT OF BURN INJURY	IMPACTED INDIVIDUALS	N
Paid more than average cost (NR 4,300/£30) for **primary** care	P, Y	2/10
Paid less than average cost (NR 4,300/£30) for **primary** care	B, J, N, S, T, Q, Z, X3	8/10
Paid more than average daily cost (NR 11,550/£80) for **secondary/tertiary** care	F, Q, R, A4	4/14
Paid less than average daily cost (NR 11,550/£80) for **secondary/tertiary** care	A, C, G, H, I, L, O, V, X, B4	10/14
Borrowed money or sold assets to pay for treatment	A, C, F, G, H, J, L, Q, R, S, T, V, B4	13/24
Reported a major loss of income or educational opportunity	A, C, G, H, L, Q, R, X3, B4	9/24
Reported a minor or no loss of income or educational opportunity	B, F, I, L, O, V, X, N, P, S, T, Y, Z, A4, Y3	15/24
Suffered disability that resulted in full dependence to perform one or more daily tasks (excludes very young children)	A, C, G, L, R, Y3	5/24
Suffered difficulties in relating with others (family and friends) after incident and treatment	C, G	2/24

KEY GIRL BOY WOMAN MAN

N = 24, WHO RECIEVED FORMAL MEDICAL TREATMENT

For peer review only - http://bmjopen.bmj.com/site/about/guidelines.xhtml

STROBE Statement—checklist of items that should be included in reports of observational studies

	Item No	Recommendation
**Title and abstract** **Page 1**	1	(*a*) Indicate the study's design with a commonly used term in the title or the abstract
		(*b*) Provide in the abstract an informative and balanced summary of what was done and what was found
**Introduction**		
Background/rationale Page 3	2	Explain the scientific background and rationale for the investigation being reported
Objectives Page 4	3	State specific objectives, including any prespecified hypotheses
**Methods**		
Study design Page 4	4	Present key elements of study design early in the paper
Setting Page 4; dates page 6	5	Describe the setting, locations, and relevant dates, including periods of recruitment, exposure, follow-up, and data collection
Participants Page 4-5	6	(*a*) *Cohort study*—Give the eligibility criteria, and the sources and methods of selection of participants. Describe methods of follow-up<br>*Case-control study*—Give the eligibility criteria, and the sources and methods of case ascertainment and control selection. Give the rationale for the choice of cases and controls<br>*Cross-sectional study*—Give the eligibility criteria, and the sources and methods of selection of participants
		(*b*) *Cohort study*—For matched studies, give matching criteria and number of exposed and unexposed<br>*Case-control study*—For matched studies, give matching criteria and the number of controls per case
Variables N/A	7	Clearly define all outcomes, exposures, predictors, potential confounders, and effect modifiers. Give diagnostic criteria, if applicable
Data sources/ measurement N/A	8*	For each variable of interest, give sources of data and details of methods of assessment (measurement). Describe comparability of assessment methods if there is more than one group
Bias N/A	9	Describe any efforts to address potential sources of bias
Study size Page 4	10	Explain how the study size was arrived at
Quantitative variables Page 6-7	11	Explain how quantitative variables were handled in the analyses. If applicable, describe which groupings were chosen and why
Statistical methods Page 6	12	(*a*) Describe all statistical methods, including those used to control for confounding
		(*b*) Describe any methods used to examine subgroups and interactions
		(*c*) Explain how missing data were addressed
		(*d*) *Cohort study*—If applicable, explain how loss to follow-up was addressed<br>*Case-control study*—If applicable, explain how matching of cases and controls was addressed<br>*Cross-sectional study*—If applicable, describe analytical methods taking account of sampling strategy
		(*e*) Describe any sensitivity analyses

For peer review only

For peer review only - http://bmjopen.bmj.com/site/about/guidelines.xhtml

**Results**		
Participants<br>Page 7	13*	(a) Report numbers of individuals at each stage of study—eg numbers potentially eligible, examined for eligibility, confirmed eligible, included in the study, completing follow-up, and analysed
		(b) Give reasons for non-participation at each stage
		(c) Consider use of a flow diagram
Descriptive data<br>Page 7	14*	(a) Give characteristics of study participants (eg demographic, clinical, social) and information on exposures and potential confounders
		(b) Indicate number of participants with missing data for each variable of interest
		(c) *Cohort study*—Summarise follow-up time (eg, average and total amount)
Outcome data<br>N/A	15*	*Cohort study*—Report numbers of outcome events or summary measures over time
		Case-control study—Report numbers in each exposure category, or summary measures of exposure
		Cross-sectional study—Report numbers of outcome events or summary measures
Main results<br>N/A	16	(*a*) Give unadjusted estimates and, if applicable, confounder-adjusted estimates and their precision (eg, 95% confidence interval). Make clear which confounders were adjusted for and why they were included
		(*b*) Report category boundaries when continuous variables were categorized
		(*c*) If relevant, consider translating estimates of relative risk into absolute risk for a meaningful time period
Other analyses<br>N/A	17	Report other analyses done—eg analyses of subgroups and interactions, and sensitivity analyses
**Discussion**		
Key results<br>Page 9	18	Summarise key results with reference to study objectives
Limitations<br>Page 10	19	Discuss limitations of the study, taking into account sources of potential bias or imprecision. Discuss both direction and magnitude of any potential bias
Interpretation<br>Page 10	20	Give a cautious overall interpretation of results considering objectives, limitations, multiplicity of analyses, results from similar studies, and other relevant evidence
Generalisability<br>Page 10	21	Discuss the generalisability (external validity) of the study results
**Other information**		
Funding<br>Page 11	22	Give the source of funding and the role of the funders for the present study and, if applicable, for the original study on which the present article is based

*Give information separately for cases and controls in case-control studies and, if applicable, for exposed and unexposed groups in cohort and cross-sectional studies.

Note: An Explanation and Elaboration article discusses each checklist item and gives methodological background and published examples of transparent reporting. The STROBE checklist is best used in conjunction with this article (freely available on the Web sites of PLoS Medicine at http://www.plosmedicine.org/, Annals of Internal Medicine at http://www.annals.org/, and Epidemiology at http://www.epidem.com/). Information on the STROBE Initiative is available at www.strobe-statement.org.

For peer review only

For peer review only - http://bmjopen.bmj.com/site/about/guidelines.xhtml
